# *Citrobacter rodentium* induces rapid and unique metabolic and inflammatory responses in mice suffering from severe disease

**DOI:** 10.1111/cmi.13126

**Published:** 2019-10-30

**Authors:** Danielle Carson, Rachael Barry, Eve G.D. Hopkins, Theodoros I. Roumeliotis, Diego García‐Weber, Caroline Mullineaux‐Sanders, Eran Elinav, Cécile Arrieumerlou, Jyoti S. Choudhary, Gad Frankel

**Affiliations:** ^1^ Centre for Molecular Microbiology and Infection, Department of Life Sciences Imperial College London London UK; ^2^ Functional Proteomics Group, Chester Beatty Laboratories Institute of Cancer Research London UK; ^3^ Inserm U1016 Institute Cochin Paris France; ^4^ CNRS UMR 8104 Paris France; ^5^ Sorbonne Paris Cité Université Paris Descartes Paris France; ^6^ Department of Immunology Weizmann Institute of Science Rehovot Israel

**Keywords:** *Citrobacter rodentium*, immunology, infection, metabolic processes, microbial‐cell interaction

## Abstract

The mouse pathogen *Citrobacter rodentium* is used to model infections with enterohaemorrhagic and enteropathogenic *Escherichia coli* (EHEC and EPEC). Pathogenesis is commonly modelled in mice developing mild disease (e.g., C57BL/6). However, little is known about host responses in mice exhibiting severe colitis (e.g., C3H/HeN), which arguably provide a more clinically relevant model for human paediatric enteric infection. Infection of C3H/HeN mice with *C. rodentium* results in rapid colonic colonisation, coinciding with induction of key inflammatory signatures and colonic crypt hyperplasia. Infection also induces dramatic changes to bioenergetics in intestinal epithelial cells, with transition from oxidative phosphorylation (OXPHOS) to aerobic glycolysis and higher abundance of SGLT4, LDHA, and MCT4. Concomitantly, mitochondrial proteins involved in the TCA cycle and OXPHOS were in lower abundance. Similar to observations in C57BL/6 mice, we detected simultaneous activation of cholesterol biogenesis, import, and efflux. Distinctly, however, the pattern recognition receptors NLRP3 and ALPK1 were specifically induced in C3H/HeN. Using cell‐based assays revealed that *C. rodentium* activates the ALPK1/TIFA axis, which is dependent on the ADP‐heptose biosynthesis pathway but independent of the Type III secretion system. This study reveals for the first time the unfolding intestinal epithelial cells' responses during severe infectious colitis, which resemble EPEC human infections.

## Graphical abstract

The outcome of *Citrobacter rodentium* infection is dependent on the genetic background of the host, with C57BL/6 and C3H/HeN mice developing self‐limiting and fatal colitis, respectively. In this paper, Carson et al have demonstrated accelerated colonisation and onset of conserved infection signatures in C3H/HeN mice. Uniquely to C3H/HeN mice,the expression of the pattern recognition receptors NLRP3 and ALPK1, as well as lactate fermentation associated proteins, LDHA and MCT4, is induced in infected intestinal epithelial cells.

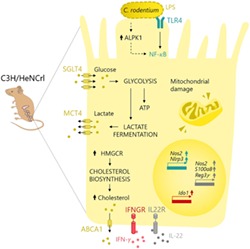

## 1 INTRODUCTION

*Citrobacter rodentium* is a mouse‐restricted extracellular pathogen commonly used to model pathogen–host interactions during enterohaemorrhagic and enteropathogenic *Escherichia coli* (EHEC and EPEC) infection and inflammatory bowel disease (IBD; Collins et al., 2014; Mullineaux‐Sanders et al., 2019; Mundy, MacDonald, Dougan, Frankel, & Wiles, 2005). The outcome of *C. rodentium* infection is dependent on the genetic background of the host. Whereas C57BL/6 mice develop a mild self‐limiting infection, C3H mice (e.g., C3H/HeN, which encodes intact TLR4) develop severe fatal diarrhoeal disease, which arguably provides a more clinically relevant model for human paediatric diarrhoeal disease, where enteropathogenic *Escherichia coli* (EPEC) infection can be lethal (Liu et al., 2016). However, due to the availability of mouse resources (e.g., knockout mice), most of our current knowledge of *C. rodentium* infection comes from studies in C57BL/6 mice, whereas little is currently known about pathogen–host interactions in mice developing severe disease.

The infection cycle of *C. rodentium* in C57BL/6 mice is divided into four defined phases (Mullineaux‐Sanders et al., 2019). An initial establishment phase (1–3 days post infection [DPI]), where *C. rodentium* resides in the caecal patch, is followed by an expansion phase (4–8 DPI) in which *C. rodentium* colonises the colon, initially adhering sporadically to the upper surface of the crypts and then expanding along the entire colonic mucosa. *C. rodentium* reaches peak bacterial load and the beginning of steady‐state phase at 8 DPI, and the bacterial clearance phase begins at 12 DPI. During the clearance phase, elimination of *C. rodentium* from the mucosa is mediated by serum IgG and phagocytosis, whereas in the lumen, the pathogen is outcompeted by commensals (Kamada et al., 2015; Masuda et al., 2008). In contrast, C3H/HeN mice are unable to clear *C. rodentium* infection, which causes significant weight loss and dehydration, with animals reaching their humane endpoint at 10–12 DPI (Vallance, Deng, Jacobson, & Finlay, 2003).

Infection of both C57BL/6 and C3H/HeN mice is dependent on a Type III secretion system (T3SS), which injects bacterial proteins directly into the cytosol of intestinal epithelial cells (IECs), where they subvert multiple signalling pathways (Pinaud, Sansonetti, & Phalipon, 2018; Wong et al., 2011). One of the hallmarks of *C. rodentium* infection is induction of attaching and effacing lesions, which are characterised by effacement of the brush border microvilli and intimate attachment of the pathogen to the apical surface of IECs (Mundy et al., 2005). A second hallmark of *C. rodentium* infection is colonic crypt hyperplasia (CCH), a tissue regeneration response manifested by proliferation of IECs (Berger et al., 2018; Collins et al., 2014). IECs comprise a number of different cell types that form a defensive barrier that is replenished every 3 to 5 days (Barker, 2014). Cell renewal is fuelled by the canonical self‐regulating signalling pathways that maintain gut homeostasis including Wnt, ZNRF3, and its paralogue RNF43 (Beumer & Clevers, 2016; Gehart & Clevers, 2019; Hao et al., 2012). At the base of the colonic crypts lie deep crypt secretory cells and LGR5+ stem cells, which give rise to a zone of rapidly dividing transit amplifying cells that terminally differentiate into absorptive enterocytes, goblet cells, enteroendocrine cells, and tuft cells (Gehart & Clevers, 2019; Johansson & Hansson, 2016; Sasaki et al., 2016). *C. rodentium‐*induced CCH is fuelled by *Rspo3‐*mediated Wnt signalling in both C57BL/6 and C3H/HeN mice. In contrast, *Rspo2* is specifically induced in C3H mice, amplifying Wnt signalling and leading to excessive expansion of transit amplifying cells, which are poorly differentiated, characterised by low abundance of the colonocyte markers SLC26A3 and CA4 responsible for chloride exchange and fluid absorption (Diez et al., 2011; Kang et al., 2018; Kang, Yousefi, & Gruenheid, 2016; Papapietro et al., 2013; Teatero et al., 2011).

Moreover, colitis susceptibility loci termed *Helicobacter hepaticus‐*induced colitis (*Hiccs*) and cytokine‐deficiency induced colitis susceptibility (*Cdcs1*) were identified on the genomes of mouse strains suffering from severe colitis, including the C3H derivative C3H/HeJBir (Bleich et al., 2010; Borm et al., 2005; Boulard, Kirchberger, Royston, Maloy, & Powrie, 2012; Farmer et al., 2001). The core region of these loci, which controls cytokine expression and innate inflammatory responses, is essentially identical (Boulard et al., 2012; Ryzhakov et al., 2018). The *Cdcs1/Hiccs* locus encodes the pattern recognition receptor alpha‐protein kinase 1 (ALPK1), which is activated by ADP‐D/L‐*glycero*‐β‐D‐*manno*‐heptose (ADP‐hep) from Gram negative bacteria, including *E. coli*, leading to oligomerisation of TRAF‐interacting protein with forkhead associated domain (TIFA), an adaptor protein involved in TNFR/TLR/NF‐κB and NLRP3 inflammasome signalling (Garcia‐Weber et al., 2018; Pfannkuch et al., 2019; Zhou et al., 2018). During the establishment phase of *C. rodentium* infection in C57BL/6 mice, NF‐κB and Caspase‐11, comprising the non‐canonical inflammasome that binds cytosolic LPS, are activated, leading to secretion of proinflammatory cytokines (e.g., CXCL1 and IL‐18) and neutrophil recruitment (Pallett et al., 2017). Moreover, C57BL/6 mice lacking the inflammasome components NLRP3, ASC, and NLRC4 exhibit increased *C. rodentium* load and intestinal inflammation, attributed to a lack of NLRP3 and NLRC4 activation in nonhaematopoietic cells (Nordlander, Pott, & Maloy, 2014; Song‐Zhao et al., 2014).

Recently, we reported temporal changes to CCH, bioenergetics, metabolism, and IL‐22 responses (e.g., nutritional immunity) in IECs during the establishment and expansion phases of *C. rodentium* infection in C57BL/6 mice. In particular, we observed drastic changes to mitochondrial processes, with a transition from oxidative phosphorylation (OXPHOS) to aerobic glycolysis, and simultaneous activation of cholesterol efflux (e.g., ABCA1) and biogenesis/import (e.g., HMGCR/LDLR), which are controlled by the transcription factors LXR/RXR and SREBP2, respectively (Berger et al., 2017; Hopkins, Roumeliotis, Mullineaux‐Sanders, Choudhary, & Frankel, 2019; Mullineaux‐Sanders et al., 2019). Importantly, both NLRP3 and TIFA have been shown to be SREBP2 transcriptional targets (Lin et al., 2016).

In this study, we applied a combination of in vivo imaging, ‐omics analysis, and cell biology to study host and microbiome responses to *C. rodentium* infection in C3H/HeN mice. This revealed rapid and uniform colonisation of *C. rodentium* at 3 DPI, 3 days earlier than in mice suffering from mild disease (C57BL/6). Earlier colonisation coincided with robust induction of host responses as early as 2 DPI and distinct upregulation of a unique repertoire of metabolic and immune related genes, including LDHA, MCT4, NLRP3, and ALPK1, specifically in C3H/HeN IECs.

## 2 RESULTS

### 2.1 *C. rodentium* establishes rapid colonic infection in C3H/HeN mice

**Figure 1:**
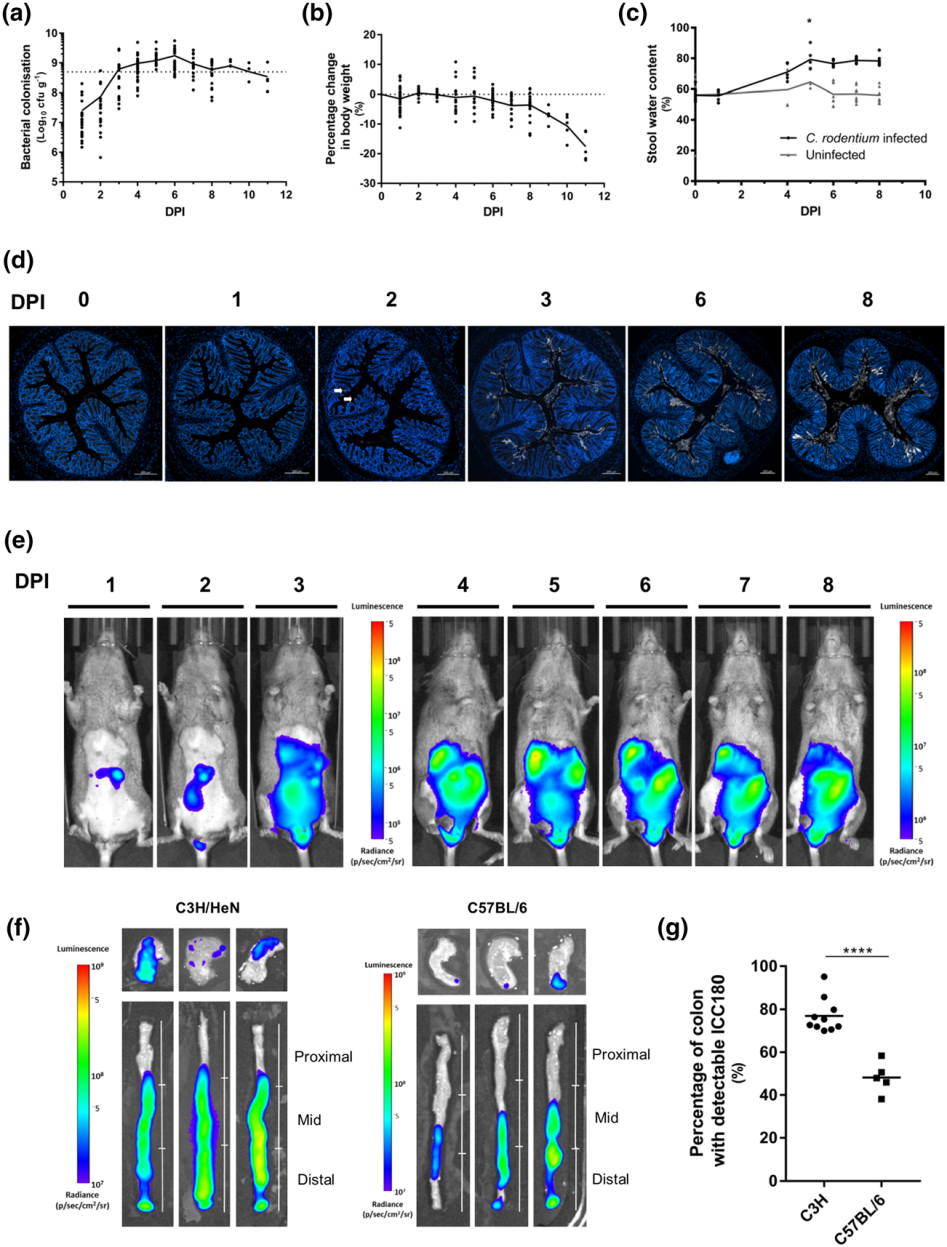
*Citrobacter rodentium* infection in C3H/HeN mice. Temporal faecal shedding (a), changes to body weight (b), and increased faecal water content (c) in individual infected mice. Solid connecting line indicates the mean. ^*^ *P* < .05 (Students *t* test). (d) Representative colonic sections from C3H/HeN mice stained for *C. rodentium* (white) and DNA (blue) at 0, 1, 2, 3, 6, and 8 DPI (*n* ≥ 4). Sporadic colonisation is seen at 2 DPI (arrows); uniform colonisation is seen from 3 DPI. Scale bar 200 μm. (e) Representative in vivo images of C3H/HeN mice infected with *C. rodentium* ICC180 showing caecal colonisation at 1 DPI and robust colonic colonisation from 3 DPI (*n* ≥ 7). (f) Ex vivo tissue images of three representative C3H/HeN (*n* = 10) and C57BL/6 mice (*n* = 5) at 8 DPI, showing extended colonic colonisation of the former. The scale bar in e and f indicates signal intensity (photons s^−1^ cm^−2^ sr^−1^). (g) Quantification of the colon length colonised by *C. rodentium* as depicted in (f). ^****^ *P* < .0001 (Students *t* test)

We monitored colonisation and the health status of the host following oral gavage of C3H/HeN mice with the bioluminescent *C. rodentium* strain ICC180. Enumeration of faecal colony‐forming units (CFU) revealed that *C. rodentium* shedding reached levels of 5 × 10^8^ CFU g^−1^ stool by 3 DPI and peaked at 2 × 10^9^ CFU g^−1^ stool at 6 DPI (Figure 1a). From 6 DPI, shedding plateaued, coinciding with signs of morbidity, including weight loss and increased faecal water content (Figure 1b,c). All mice reached the predefined endpoint by 11 DPI. Analysis of temporal colonic colonisation by staining detected low and sporadic levels of *C. rodentium* on the distal colonic mucosa from 2 DPI and uniform colonisation at 3 DPI, which was maintained until 8 DPI (Figure 1d). For comparison, uniform colonisation of C57BL/6 mice was only observed from 6 DPI (Hopkins et al., 2019).

We used live in vivo bioluminescent imaging (BLI) to compare infection dynamics of C3H/HeN and C57BL/6 mice. This revealed caecal colonisation of C3H/HeN mice at 1 DPI with extensive colonisation of both the caecum and colon at 3 DPI, which was maintained until 8 DPI (Figure 1e). In contrast, following a longer establishment phase in the caecum (1–4 DPI), colonisation in C57BL/6 mice was largely confined to the colon from 5 DPI with significantly less bioluminescent signal in the caecum and colon (Figure S1A–C; Hopkins et al., 2019; Wiles, Pickard, Peng, MacDonald, & Frankel, 2006). Ex vivo BLI revealed that ICC180 had colonised the caecal tissue and the mid and distal colon, extending into the proximal colon of C3H/HeN mice (Figure 1f). Colonisation covered 77.0 ± 7.9% of the colon length at 8 DPI (Figure 1f,g), significantly higher than the 48.2 ± 7.3% observed in C57BL/6 mice at the same time point. Overall, these data suggest that compared with C57BL/6 mice, the pathophysiology of C3H/HeN mice is mediated by earlier *C. rodentium* colonisation with increased coverage of the colonic and caecal tissue.

### 2.2 The epithelial proteome of C3H/HeN mice responds swiftly to *C. rodentium*

To study global changes in the biological processes of C3H/HeN IECs in response to *C. rodentium* infection, we carried out quantitative mass spectrometry‐based proteomic analysis. IEC proteins were extracted from at least four mice colonised to levels greater than 1 × 10^8^ CFU g^−1^ of stool at 6 DPI. Protein lysate of two biological repeats were pooled at a 1:1 ratio and used to quantify infection‐induced fold change (FC) in protein abundance compared with an uninfected control (Figure S2). As a control, we simultaneously analysed the C57BL/6 IECs' proteome at 6 DPI. Comparative gene set enrichment analysis of C3H/HeN and C57BL/6 revealed largely conserved changes to IEC processes upon infection (Figure S3). The KEGG pathways, *Staphylococcus aureus* infection, complement and coagulation cascades, and phagosome were found to be opposingly regulated at 6 DPI; however, the differential response of proteins assigned to these pathways converged by 8 DPI (PRIDE identifier: PXD005004; Berger et al., 2017). A comprehensive analysis of the C57BL/6 proteome has recently been reported elsewhere (Berger et al., 2017; Hopkins et al., 2019).

**Figure 2:**
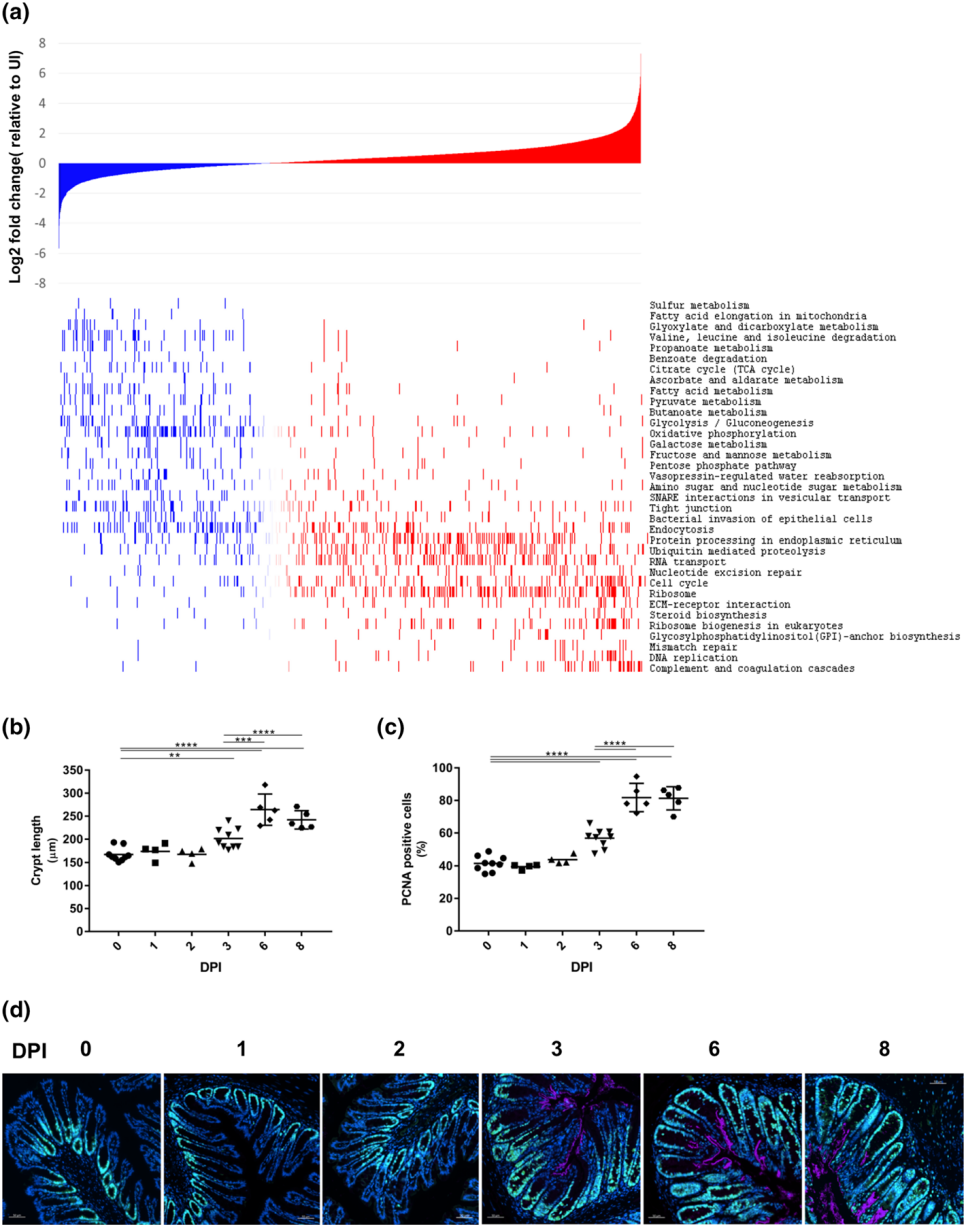
*Citrobacter rodentium* subverts metabolism and cell proliferation in C3H/HeN IECs. (a) Log2 fold change values of proteins ranked from most downregulated (left) to most upregulated (right) in abundance in infected samples compared with uninfected (top panel). KEGG pathways determined as significantly enriched following 1D enrichment analysis of the infected IEC proteome compared with uninfected samples and ranked from most negatively enriched (i.e., most downregulated; top) to most positively enriched pathways (i.e., most upregulated; bottom). Individual proteins associated with ranked KEGG pathways are highlighted in the heat‐map adjacent to the corresponding KEGG pathway (lower panel). (b) Crypt length measurements over time showing increased CCH at 3 DPI followed by a further expansion at 6 DPI (each point represents the mean crypt length of an individual mouse). (c) Quantification of the PCNA‐positive zone calculated as a percentage of the total crypt length (each point represents the mean crypt length of an individual mouse). (d) Representative immunostaining of PCNA (green), *C. rodentium* (pink), and DNA (blue) from *C. rodentium‐*infected colonic sections, showing an increased proliferation zone at 3 DPI followed by a further expansion at 6 DPI (*n* ≥ 4). Scale bar 50 μm

We quantified a total of 11,602 proteins in C3H/HeN mice, of which 9,184 were mapped to *Mus musculus* and 2,418 to *C. rodentium* genes (peptide false discovery rate [FDR] <1%). Proteins with altered abundance at or above 1.5‐fold (0.59 Log2 FC) compared with the uninfected control samples were considered changed upon infection. Gene set enrichment analysis revealed mitochondrial associated processes such as fatty acid elongation (enrichment score: −0.74, *P* value: .003), TCA cycle (enrichment score: −0.59, *P* value: 8.29E‐11), and OXPHOS (enrichment score: −0.46, *P* value: 2.51E‐15) amongst the most downregulated pathways (Figure 2a). Conversely, steroid biosynthesis (enrichment score: 0.44, *P* value: 0.003), DNA replication (enrichment score: 0.61, *P* value: 1.14E‐08), and cell cycle (enrichment score: 0.31, *P* value: 9.96E‐07) associated proteins were found in significantly higher abundance in infected IECs (Figure 2a).

As pathway analysis revealed significant enrichment of processes involved in cell proliferation, we quantified the temporal induction of CCH in infected C3H/HeN mice. We found that CCH was induced in two stages: an initial expansion was observed at 3 DPI, which was followed by a further increase in crypt length at 6 DPI that remained unchanged at 8 DPI (Figure 2b). This two‐step expansion was mirrored by a staggered increase in the proliferation zone, visualised by cells positive for the proliferation marker proliferating cell nuclear antigen (PCNA ; involved in DNA replication in dividing cells, Figure 2c,d). Importantly, at 6 DPI, PCNA‐positive cells composed 81.3 ± 7.1% of the total length of the crypt (Figure 2c), compared with approximately 50% previously reported in C57BL/6 mice at 6 DPI (Hopkins et al., 2019).

**Figure 3:**
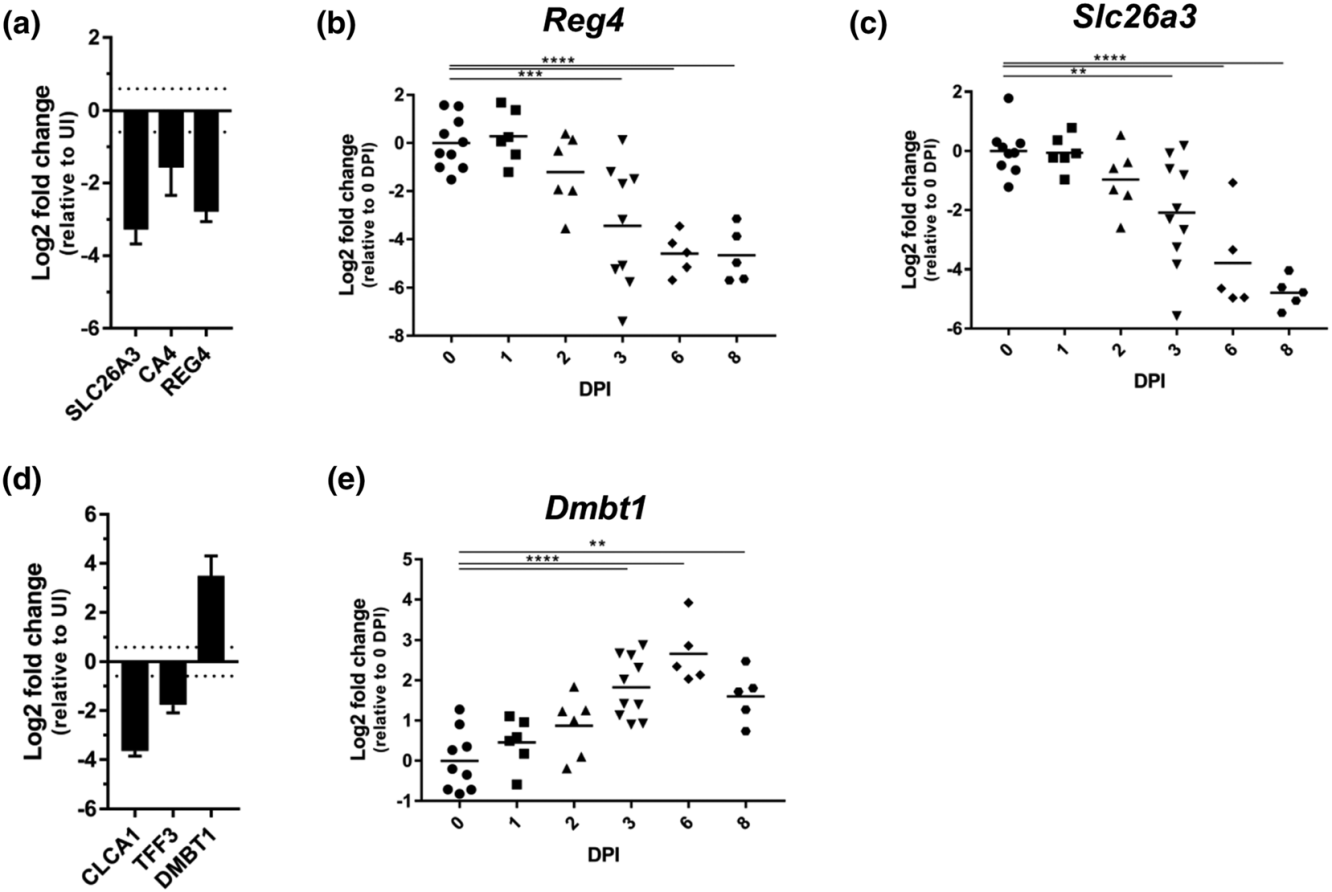
Reprogramming of the gut epithelium during *C. rodentium‐*induced CCH. The protein abundance of SLC26A3, CA4, and REG4 (a) and CLCA1 and TFF3 (d) decreases, whereas that of DMBT1 increases upon infection (d). Bars indicate standard deviation. The abundance of *Slc26a3* (b), *Reg4* (c), and *Dmbt1* (e) transcripts show a decrease in *Slc26a3* and *Reg4* and an increase in *Dmbt1* from 3 DPI. Each point represents an individual mouse at 0, 1, 2, 3, 6, and 8 DPI. ^*^ *P* ≤ .05, ^**^ *P* ≤ .01, ^***^ *P* ≤ .001, ^****^ *P* ≤ .0001 (one‐way ANOVA)

Consistently, the proteomic analysis revealed decreased abundance of the colonocyte cellular differentiation markers SLC26A3 (−3.32 Log2 FC) and CA4 (−1.65 Log2 FC) and the deep crypt secretory cell marker REG4 (−2.78 Log2 FC), at 6 DPI (Figure 3a). Following temporal expression of these genes by qRT‐PCR showed significantly lower *Reg4* and *Slc26a3* expression from as early as 3 DPI (Figure 3b,c), compared with 6 and 8 DPI in C57BL/6 mice for *Reg4* and *Slc26a3*, respectively (Hopkins et al., 2019). Similarly to C57BL/6 mice, the proteomic analysis revealed that the abundance of goblet cell proteins CLCA1 and TFF3 decreased in the infected IEC proteome of C3H/HeN mice by −3.65 and −1.77 Log2 FC, respectively (Figure 3d; Hopkins et al., 2019). In contrast, and in line with observations in C57BL/6 mice, DMBT1, a mucus‐associated glycoprotein that inhibits bacterial attachment to IECs, was found in higher abundance following infection (3.60 Log2 FC, Figure 3d; Rosenstiel et al., 2007); qRT‐PCR showed significant transcriptional induction of *Dmbt1* from 3 DPI (Figure 3e) earlier than observed in C57BL/6 mice (8 DPI; Hopkins et al., 2019). These results show that the epithelium of C3H/HeN mice, which experience severe disease, undergoes faster reorganisation compared with mice suffering from mild disease.

### 2.3 *C. rodentium* triggers aerobic glycolysis and lactate efflux in C3H/HeN mice

**Figure 4:**
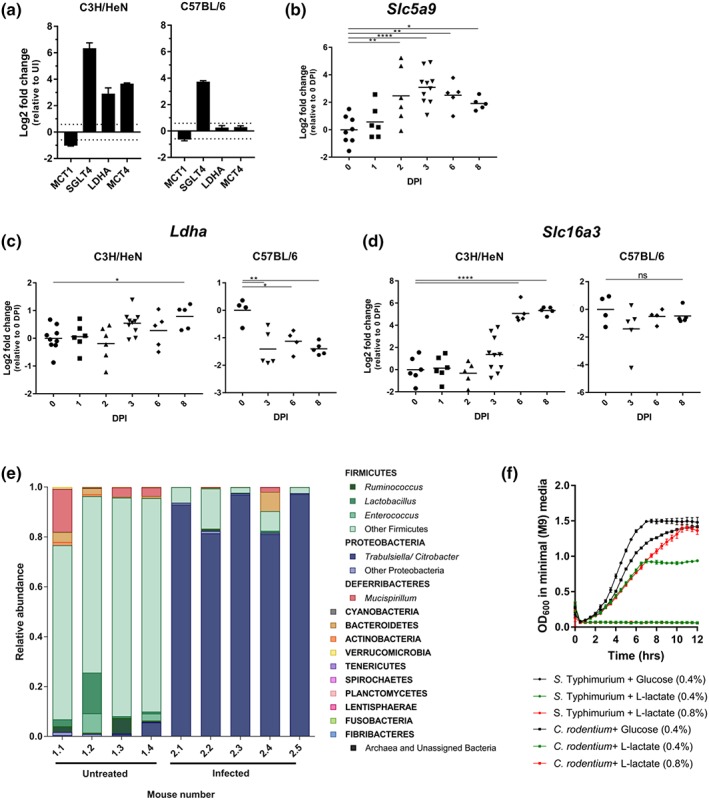
*Citrobacter rodentium* induces changes to IEC metabolism. (a) The protein abundance of MCT1 decreases and SGLT4 increases upon infection of both C3H/HeN and C57BL/6 mice. The abundance of LDHA and MCT4 increases specifically in infected C3H/HeN mice. Bars indicate standard deviation. (b) The abundance of Slc5a9 transcripts (encoding SGLT4) increases from 2 DPI. (c) The abundance of Ldha transcripts increases at 8 DPI in C3H/HeN mice and decreases from 6 DPI in C57BL/6 mice. (d) The abundance of Slc16a3 transcripts (encoding MCT4) increases from 6 DPI in C3H/HeN mice and is unchanged over time in C57BL/6 mice. For all qRT‐PCR data, each point represents an individual mouse at 0, 1, 2, 3, 6, and 8 DPI. *P ≤ .05, **P ≤ .01, ***P ≤ .001, and ****P ≤ .0001 (one‐way ANOVA). (e). Relative abundancies of tissue‐associated microbiota phyla in uninfected (n = 4) and *C. rodentium*‐infected mice (n = 5), showing a bloom of proteobacteria and depletion of Firmicutes upon infection. *P < .05 (Mann–Whitney). (f) *S.* Typhimurium, but not *C. rodentium*, can utilise L‐lactate as a sole carbon source. Both strains can grow on minimal medium containing glucose

In agreement with previously reported data, we observed increased abundance of the basolateral glucose transporter SGLT4, which feeds aerobic glycolysis and fuels cell proliferation in both C3H/HeN and C57BL/6 mice (6.37 and 3.74 Log2 FC, respectively, Figure 4a; Berger et al., 2017; Hopkins et al., 2019). Temporal qRT‐PCR analysis revealed that transcription of S*lc5a9* (encoding SGLT4) increased from 2 DPI in C3H/HeN mice, coinciding with sporadic *C. rodentium* association with the colonic mucosa (Figure 4b). In addition, we observed reduced abundance of the apical transporter MCT1 in both C3H/HeN and C57BL/6 mice (−1.03 and −0.64 Log2 FC, respectively), which is implicated in import of microbiota‐generated butyrate that feeds the TCA cycle (Berger et al., 2017).

Uniquely to C3H/HeN IECs, we observed increased abundance of lactate dehydrogenase A (LDHA; 2.94 and 0.26 Log2 FC in C3H/HeN and C57BL/6, respectively), which mediates conversion of pyruvate to lactate, and the basolateral lactate efflux transporter MCT4 (3.67 and 0.29 Log2 FC in C3H/HeN and C57BL/6, respectively; Figure 4a). Validation by qRT‐PCR revealed increased transcription of *Ldha* (encoding lactate dehydrogenase) in the IECs of C3H/HeN mice at 8 DPI, whereas in C57BL/6 mice, it decreased from 3 DPI (Figure 4c). In addition, *Slc16a3* (encoding MCT4) showed significantly increased expression from 6 DPI in C3H/HeN IECs but was unchanged in C57BL/6 (Figure 4d). Together, these results show that although many metabolic pathways are modulated in similar ways, there is a distinct strain specific regulation of several key proteins.

Similarly to infection of C57BL/6 mice with either *C. rodentium* or *Salmonella enterica* serovar Typhimurium, infection of C3H/HeN mice with *C. rodentium* resulted in reduced abundance of tissue‐associated Firmicutes, including butyrate‐producing *Clostridia*, which coincided with a bloom of Enterobacteriaceae (Figures 4e and S4a–g; Berger et al., 2017; Gillis et al., 2018; Hopkins et al., 2019). It has previously been shown that *S*. Typhimurium mediated depletion of *Clostridia* induces a switch in host epithelial cell metabolism from OXPHOS to lactate fermentation, increasing lactate availability which is subsequently utilised by *Salmonella* during infection (Gillis et al., 2018). We therefore determined whether *C. rodentium* can use L‐lactate as a carbon source, with *S*. Typhimurium as a control. Using minimal (M9) media containing L‐lactate as the sole carbon source revealed that unlike *S*. Typhimurium, *C. rodentium* was unable to grow on L‐lactate (Figure 4f). Both *S*. Typhimurium and *C. rodentium* grew equally well in M9 containing glucose used as a control (Figure 4f). These results suggest that the availability of luminal L‐lactate might contribute to the bloom of Enterobacteriaceae and that *C. rodentium* might only benefit from this indirectly.

### 2.4 *C. rodentium* induces conserved changes to cholesterol homeostasis

**Figure 5:**
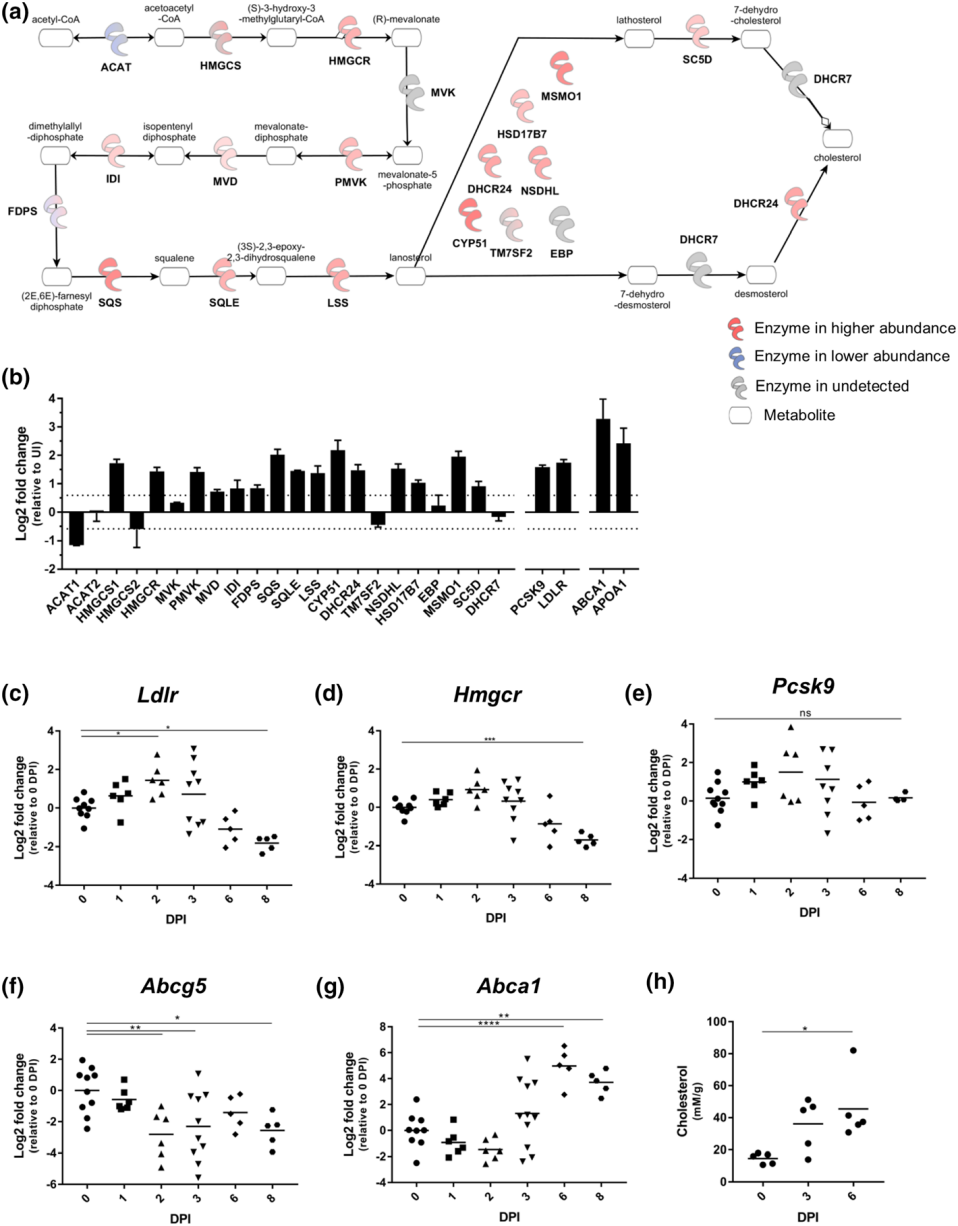
*Citrobacter rodentium* induces changes to cholesterol homeostasis. (a) Schematic showing the regulated proteins in the cholesterol biosynthetic pathway. (b) The protein abundance of the nearly all proteins in the cholesterol biosynthetic pathway increases upon *C. rodentium* infection. Bars indicate standard deviation. The abundance of *Ldlr* (c) and *Hmgcr* (d) transcripts decreases at 8 DPI, respectively, whereas *Pcsk9* is unchanged (e). The abundance of *Abcg5* decreases from 2 DPI (f), whereas that of *Abca1* increases from 6 DPI (g). Each point represents an individual mouse at 0, 1, 2, 3, 6, and 8 DPI. (h) A gradual increase in faecal cholesterol measured at 0, 3, and 6 DPI in individual mice; each point indicates an individual mouse. ^*^ *P* ≤ .05, ^**^ *P* ≤ .01, ^***^ *P* ≤ .001, and ^****^ *P* ≤ .0001 (one‐way ANOVA)

Cholesterol is an important component of de novo membrane biogenesis during cell proliferation as well as lipid rafts, which amplify downstream TLR signalling through proximity‐induced receptor interactions (Litvinov, Savushkin, & Dergunov, 2018; Silvius, 2003). We have recently reported that in C57BL/6 mice infected with *C. rodentium*, SREBP2 was activated in IECs at 8 DPI, leading to increased abundance of proteins of the cholesterol biosynthetic pathway and import, including HMGCR, LDLR, and PCSK9 (Berger et al., 2017; Hopkins et al., 2019). The proteomic data of the C3H/HeN mice also showed upregulation of the steroid biosynthetic pathway at 6 DPI (Figure 5a), including HMGCR (1.34 Log2 FC), LDLR (1.66 Log2 FC), and PCSK9 (1.63 Log2 FC; Figure 5b). However, using qRT‐PCR to follow temporal gene expression revealed that contrary to the proteome, on the transcriptional level, the mRNA levels of *Hmgcr*, *Pcsk9*, and *Ldlr* did not significantly change up to 6 DPI (although *Ldlr* showed an initial increase in transcription at 2 DPI) and decreased at 8 DPI, specifically for *Hmgcr* and *Ldlr* (Figure 5c–e), suggesting that similarly to C57BL/6 mice, the level of HMGCR, PCSK9, and LDLR protein abundance may be regulated posttranscriptionally (Hopkins et al., 2019).

In parallel, the abundance of the basolateral cholesterol transporter ABCA1, which is regulated by the transcription factor LXR/RXR, and the cholesterol binding protein, APOA1 (involved in reverse cholesterol transport), were also higher in the proteomes of infected, compared with uninfected, IECs (3.11 and 1.95 Log2 FC for ABCA1 and APOA1, respectively, Figure 5b; Murthy, Born, Mathur, & Field, 2002). Temporal gene expression profiling revealed that while the level of *Abcg5* transcript, which is also controlled by LXR/RXR and was undetected in the proteome, decreased as early as 2 DPI, expression of *Abca1* was upregulated at 6 DPI and maintained at 8 DPI (Figure 5f,g). Indeed, increased faecal cholesterol was detectable by 3 DPI, which reached significance by 6 DPI (Figure 5h). Taken together, these results show that similarly to C57BL/6 mice, cholesterol biogenesis and cholesterol efflux are simultaneously activated in the IECs of C3H/HeN mice.

### 2.5 *C. rodentium* infection triggers swift IL‐22 responses in IECs of C3H/HeN mice

**Figure 6:**
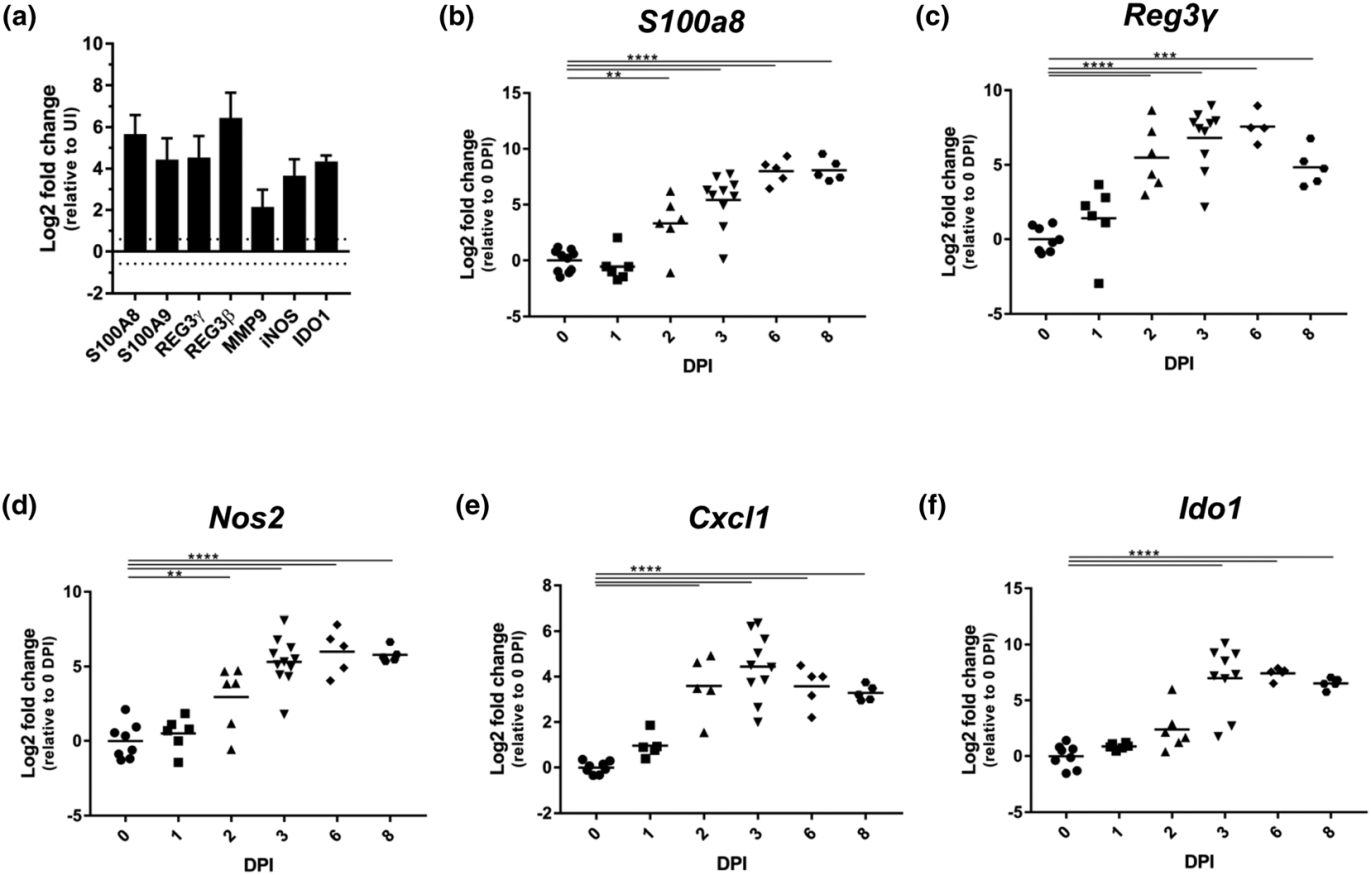
Rapid induction of IL‐22 and IFN‐γ responses occurs following *C. rodentium* infection. (a) The protein abundance of S100A8, S100A9, REG3γ, REG3β and MMP9, iNOS, and IDO1 increases upon infection (CXCL1 is undetected). Bars indicate standard deviation. The abundance of *S100a8* (b), *Reg3γ* (c), *Nos2* (d), and *Cxcl1* (e) transcripts increase from 2 DPI, whereas *Ido1* increases at 3 DPI (f). ^*^ *P* ≤ .05, ^**^ *P* ≤ 0.01, ^***^ *P* ≤ .001, and ^****^ *P* ≤ .0001 (one‐way ANOVA)

Cholesterol is an essential component of cellular membranes, needed for sustainable cell proliferation. In addition, proliferation of IECs is a tissue repair response triggered by IL‐22, which also mediates expression of nutritional immunity proteins, for example, calprotectin and antimicrobial peptides, including REG3γ and REG3β, as well as matrix metallopeptidase 9 (MMP9) and inducible nitric oxide synthase (iNOS). Consistently, the proteomics analysis revealed that the abundances of S100A8, S100A9, REG3γ and REG3β, MMP9, and iNOS were significantly increased at 6 DPI compared with uninfected controls, corresponding to 5.81, 4.60, 4.71, 6.69, 2.27, and 3.77 Log2 FC, respectively (Figure 6a). Following temporal expression of *S100a8*, *Reg3γ*, *Nos2* (encoding iNOS), and *Cxcl1* (a neutrophil recruiting chemokine undetected in the proteome) in IECs revealed significant transcriptional upregulation as early as 2 DPI, just as the pathogen is sporadically detected in the colon (Figure 6b–e). Of note, transcriptional upregulation of *S100a8*, *Reg3γ*, *Nos2*, and *Cxcl1* in C57BL/6 mice was only observed from 6 DPI (Hopkins et al., 2019). In addition, we detected a higher abundance of IDO1 (a reporter for IFN‐γ, 4.36 Log2 FC) at 6 DPI of C3H/HeN mice (Figure 6a). Elevated *Ido1* transcription was observed by 3 DPI, which was maintained at the same level up to 8 DPI (Figure 6f). These results show that while triggering similar innate immune responses in IECs, there are clear temporal differences between *C. rodentium*‐infected C3H/HeN and C57BL/6 mice, with responses occurring much earlier in the former.

### 2.6 *C. rodentium* triggers upregulation of NLRP3 and ALPK1 specifically in C3H/HeN mice

**Figure 7:**
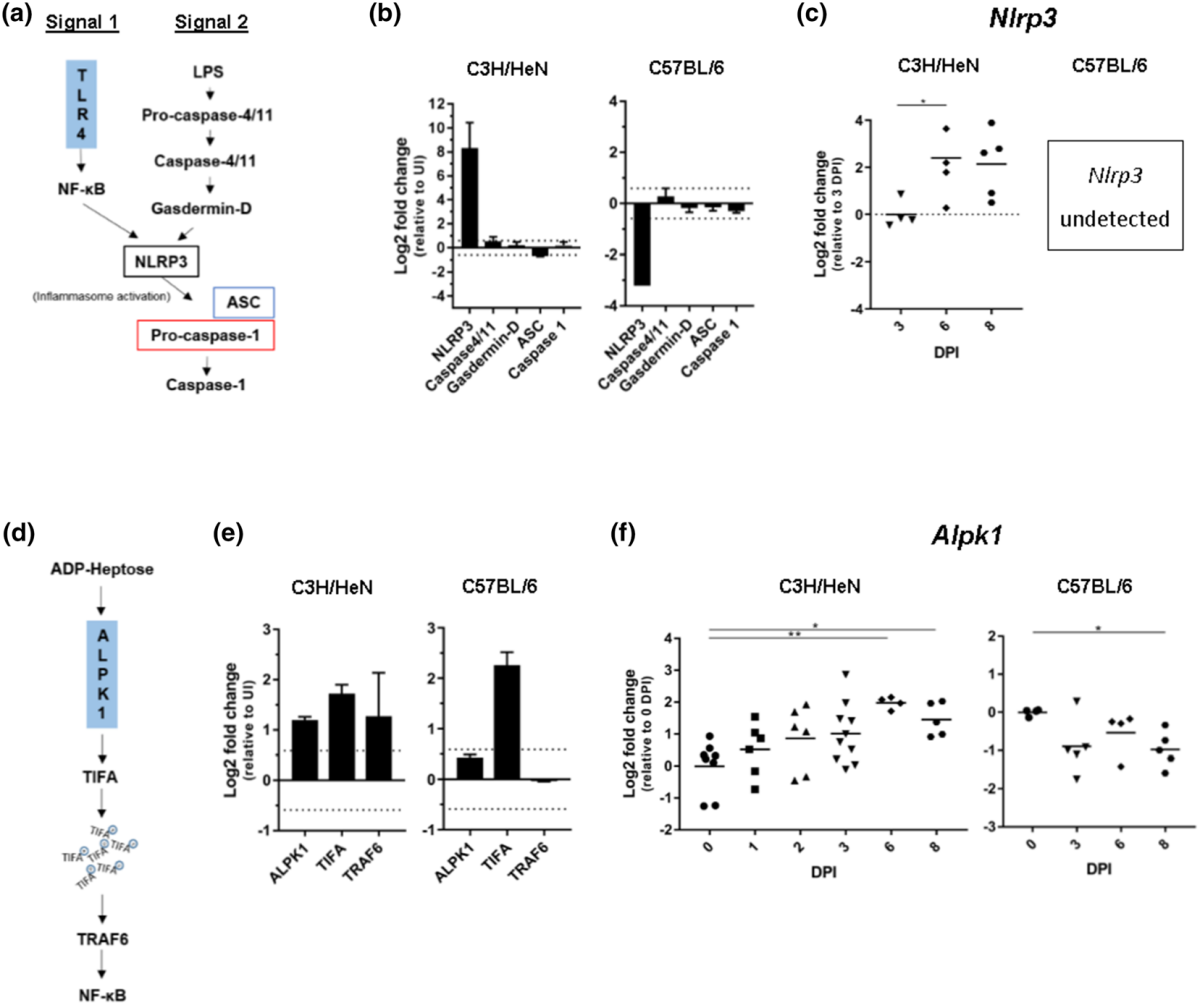
Differential regulation of ALPK1 pathway in C3H/HeN and C57BL/6 mice. (a) Schematic showing NLRP3 inflammasome (a) and ALPK1 (d) activation pathways. (b) The protein abundance of NLRP3 increases in infected C3H/HeN mice and decreases in C57BL/6. The abundance of the other inflammasome components (excluding ASC in C3H/HeN mice) do not change upon infection. Bars indicate standard deviation. (c) The abundance of *Nlrp3* transcript increases in C3H/HeN mice from 6 DPI but was undetectable in IECs from C57BL/6. (e) The protein abundance of ALPK1, TIFA, and TRAF6 increases upon *C. rodentium* infection in C3H/HeN, whereas only TIFA is increased in C57BL/6 mice. Bars indicate standard deviation. (f) The abundance of *Alpk1* transcript increases in C3H/HeN from 6 DPI, whereas expression of *Alpk1* in C57BL/6 is unchanged upon infection. For all qRT‐PCR data, each point represents an individual mouse at 0, 1, 2, 3, 6, and 8 DPI. ^*^ *P* ≤ .05, ^**^ *P* ≤ .01, ^***^ *P* ≤ .001, and ^****^ *P* ≤ .0001 (one‐way ANOVA)

Unexpectedly, NLRP3 was found in a higher abundance in the proteome of infected IECs from C3H/HeN mice (8.90 Log2 FC), whereas it was decreased in C57BL/6 (−3.21 Log2 FC). Other components of the NLRP3 inflammasome including Caspase 4/11, Gasdermin‐D, Caspase 1, and ASC were unchanged following infection in C3H/HeN (0.55, 0.13, 0.10, and −0.63 Log2 FC, respectively) and C57BL/6 mice (0.28, −0.17, −0.15, and −0.29 Log2 FC, respectively), apart from ASC which decreased in C3H/HeN (Figure 7a,b). Subsequent validation by qRT‐PCR confirmed significant transcriptional upregulation of *Nlrp3* at 6 and 8 DPI in C3H/HeN compared with 3 DPI (*Nlrp3* mRNA was undetected in uninfected mice and on 1 and 2 DPI); *Nlrp3* was undetected at all time points tested in C57BL/6 (Figure 7c).

Intriguingly, the abundance of ALPK1 increased (1.20 Log2 FC) in infected IECs from C3H/HeN mice (Figure 7d,e). The ALPK1 downstream substrates, TIFA and TRAF6, were also found in increased abundance upon infection (1.72 and 1.28 Log2 FC, respectively, Figure 7e). In contrast, the abundance of ALPK1 and TRAF6 was unchanged during infection in C57BL/6 mice (0.43 and −0.04 Log2 FC, respectively), whereas the abundance of TIFA increased (2.26 Log2 FC, Figure 7e). Temporal analysis of *Alpk1* expression by qRT‐PCR revealed gradually increasing expression in C3H/HeN mice from 1 DPI, which reached significance at 6 DPI and was maintained at 8 DPI (Figure 7f); expression of *Alpk1* remained unchanged at 3 and 6 DPI C57BL/6 mice and significantly decreased at 8 DPI (Figure 7f).

### 2.7 *C. rodentium* activates the ALPK1 pathway in a T3SS‐independent manner

Activation of the ALPK1 immune pathway has been linked to the LPS metabolite ADP‐hep (Garcia‐Weber et al., 2018; Pfannkuch et al., 2019; Zhou et al., 2018). To determine if the ALPK1 pathway is activated during *C. rodentium* infection, we first generated *C. rodentium* Δ*hldE* and Δ*rfaC* mutants. HldE, together with GmhA and GmhB, is responsible for the synthesis of ADP‐Hep from D‐sedoheptulose 7‐phosphate (S7P) through a number of intermediates (see schematic in Figure S5A). RfaC acts downstream of ADP‐hep production to facilitate transfer to 3‐deoxy‐D‐*manno*‐octulosonic acid‐lipid A (Kdo2‐lipid A); thus, we used the Δ*rfaC* mutant as a “deep rough” LPS control. As mutations in Δ*hldE* and Δ*rfaC* affect the integrity of the outer membrane, we tested if this impacted on growth. Using both rich (LB) and minimal (M9) media, we found that both mutations caused significant growth attenuation in minimal media (Figure S5B,C) and thus could not be analysed in vivo.

**Figure 8:**
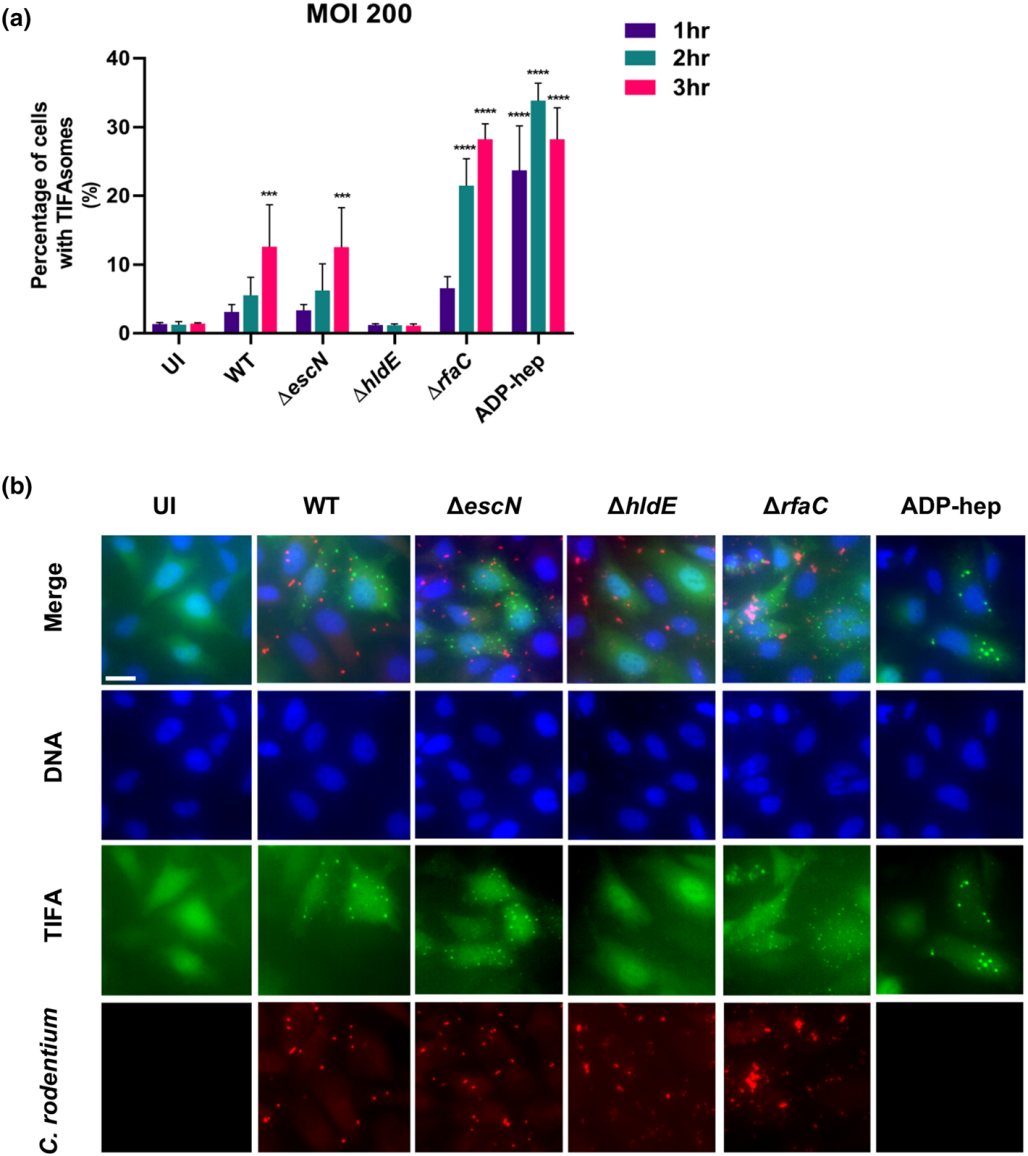
*Citrobacter rodentium* ADP‐hep metabolite induces activation of the ALPK1 pathway. (a) Quantification of TIFAsomes as depicted in (b) following 1, 2, or 3‐hr infection at MOI 200 with WT, Δ*escN*, Δ*hldE*, and Δ*rfaC C. rodentium*; treatment with ADP‐hep (10^−7^ M) was used as a control. WT *C. rodentium* and *C. rodentium* Δ*escN* similarly activates ALPK1; *C. rodentium* Δ*hldE* does not trigger TIFAsome formation whereas *C. rodentium* Δ*rfaC* triggers excessive TIFAsomes. Data correspond to the mean and standard deviation of three experiments (^***^ *P* ≤ .001 and ^****^ *P* ≤ 0.0001 [two‐way ANOVA]). (b) Representative images of HeLa‐TIFA‐GFP cells showing TIFAsomes or not following infection with WT, Δ*escN*, and Δ*hldE*, *C. rodentium* (MOI 200), or treatment with ADP‐hep. Blue, DNA; green, TIFA‐GFP; and red, *C. rodentium.* Scale bar 20 μM

We therefore determined if *C. rodentium* can activate ALPK1 by analysing formation of TIFA oligomers, also called “TIFAsomes,” following infection of TIFA‐GFP expressing reporter HeLa cells with wild type (WT), *C. rodentium* Δ*escN* (T3SS deficient), Δ*hldE*, and Δ*rfaC*; treatment with ADP‐hep (10^−7^ M) was used as a positive control. This revealed that infection with WT and Δ*escN* similarly activated ALPK1, determined by quantification of “TIFAsomes,” whereas no activation was seen by the Δ*hldE*, and as expected (due to accumulation of LPS metabolites), overactivation was seen by the Δ*rfaC* mutant (Figures 8a,b and S6). Taken together, these results suggest that *C. rodentium* triggers expression of *Alpk1* in vivo and can activate the ALPK1‐TIFA signalling axis in a T3SS‐independent manner in vitro.

## 3 DISCUSSION

Our investigation into host responses to *C. rodentium* infection in susceptible C3H/HeN mice revealed accelerated colonisation and rapid IEC responses, starting from 2 DPI, and upregulation of ALPK1 and NLRP3. In contrast, the IECs of C57BL/6 mice are nonresponsive to *C. rodentium* until 4 DPI (Hopkins et al., 2019) with no detectable upregulation of ALPK1 and NLRP3. A summary of the main differences between mice suffering from mild and severe *C. rodentium* infection is shown in Table 1.

**Table 1:** Comparison of *Citrobacter rodentium* infection signatures in severe and mild disease models

Host process	Infection signature	Marker	C3H/HeN (severe)	C57BL/6 (mild)
Bacterial colonisation	Transition between the caecal establishment and colonic expansion phase	Sporadic colonisation of colonic epithelium	2 DPI	4 DPI (Hopkins et al., 2019)
	Steady‐state infection phase	Uniform colonisation of colonic epithelium	3 DPI	6 DPI (Hopkins et al., 2019)
		CFU over 5 × 10^8^ CFU g^−1^ stool	3 DPI	6 DPI (Hopkins et al., 2019)
CCH	IL‐22 (Berger et al., 2018) and R‐spondin2/3 signalling (Papapietro et al., 2013)	Increased proliferation zone	3 DPI	4 DPI (Hopkins et al., 2019)
		Increased total crypt length	3 DPI	6 DPI (Hopkins et al., 2019)
Innate immune response	IL‐22 signalling (Zheng et al., 2008)	Increased *S100a8* expression	2 DPI	6 DPI (Hopkins et al., 2019)
		Increased *Reg3γ* expression	2 DPI	4 DPI (Hopkins et al., 2019)
	IL‐22 (Andoh et al., 2005; Wang et al., 2017; Ziesche, Bachmann, Kleinert, Pfeilschifter, & Muhl, 2007) and NF‐κB signalling (Burke et al., 2014; Malladi, Puthenedam, Williams, & Balakrishnan, 2004)	Increased *Nos2* expression	2 DPI	6 DPI (Hopkins et al., 2019)
		Increased *Cxcl1* expression	2 DPI	6 DPI (Hopkins et al., 2019)
	IFN‐γ signalling (Saha, Jyothi Prasanna, Chandrasekar, & Nandi, 2010)	Increased *Ido1* expression	3 DPI	8 DPI (Hopkins et al., 2019)
	NF‐κB (Bauernfeind et al., 2009) and SREBP‐2/TIFA signalling (Lin et al., 2016)	Increased *Nlrp3* expression	6 DPI	Undetected
	Priming of the ALPK1‐TIFA signalling axis	Increased *Alpk1* expression	6 DPI	Decrease at 8 DPI
Mucosal response	Host defence mechanism against bacterial attachment (Rosenstiel et al., 2007)	Increased *Dmbt1* expression	3 DPI	8 DPI (Hopkins et al., 2019)
Epithelial response	Loss of differentiated deep crypt secretory cells (Sasaki et al., 2016)	Decreased *Reg4* expression	3 DPI	6 DPI (Hopkins et al., 2019)
	Loss of differentiated enterocytes (Kang et al., 2016; Silberg, Wang, Moseley & Traber, 1995)	Decreased *Slc26a3* expression	3 DPI	8 DPI (Hopkins et al., 2019)
Metabolism	Lactate fermentation (Doherty & Cleveland, 2013)	Increased *Ldha* expression	8 DPI	Decreased at 3 DPI
		Increased *Slc16a3* expression	6 DPI	Unchanged

Global analysis of changes to the infected C3H/HeN and C57BL/6 IEC proteomes revealed downregulation of central metabolism processes occurring in parallel to upregulation of pathways involved in cell proliferation as well as DNA replication and DNA damage repair response. We have recently shown in C57BL/6 mice an increased PCNA‐positive proliferation zone at 4 DPI, which is followed by an increase in total crypt length at 6 DPI (Hopkins et al., 2019). In contrast, C3H/HeN mice exhibit both increased PCNA staining and CCH at 3 DPI, which were further amplified at 6 DPI. This may reflect increased R‐spondin 2 activation of Wnt/β‐catenin signalling or rapid induction of IL‐22 responses following *C. rodentium* colonisation of C3H/HeN mice.

Infection with *C. rodentium* was associated with a bloom of facultative anaerobic Enterobacteriaceae and depletion of resident flora largely composing of Firmicutes including the butyrate‐producing class *Clostridia*. These responses are conserved with *C. rodentium*‐infected (Berger et al., 2017; Hopkins et al., 2019) and *S*. Typhimurium‐infected (Gillis et al., 2018) C57BL/6 mice. Previous studies have shown that depletion of commensal *Clostridia* during *S*. Typhimurium infection triggers lactate fermentation and release of L‐lactate from the host cells. The increased levels of lactate are utilised by *S*. Typhimurium as a nutrient during infection. Specifically, in C3H/HeN mice, we found that LDHA, which mediates pyruvate conversion to lactate, and MCT4, which shuttles lactate across the cell membrane, were significantly upregulated at 6 and 8 DPI, respectively. However, unlike *S*. Typhimurium, *C. rodentium* was unable to utilise L‐lactate as a carbon source. Nonetheless, in both C3H/HeN and C57BL/6 mice, the apical butyrate transporter MCT1, which feeds OXPHOS, is downregulated whereas SGLT4, which supports aerobic glycolysis, is upregulated. This suggests that oxygenation of the colon, facilitated by the metabolic switch to glycolysis in IECs, may benefit both *C. rodentium* (which has a preference for aerobic metabolism) and the host (e.g., by boosting growth of L‐lactate catabolising Enterobacteriaceae; Garvie, 1980; Lopez et al., 2016). In contrast, it appears that *S*. Typhimurium has adapted to the L‐lactate‐rich ecological niche (Gillis et al., 2018).

Cholesterol biogenesis is a key pathway altered in IECs upon *C. rodentium* infection in both C3H/HeN (this study) and C57BL/6 mice (Berger et al., 2017; Hopkins et al., 2019). Our current understanding of the role cholesterol plays in innate immunity mainly comes from studies of macrophages, where a positive feedback loop amplifies inflammation. LXR/RXR activation represses NF‐κB signalling and expression of inflammatory proteins in macrophages, including iNOS, IL‐6, and MMP9 (Castrillo, Joseph, Marathe, Mangelsdorf, & Tontonoz, 2003). However, iNOS and MMP9 were amongst the proteins with the highest increased abundance in IECs during infection in both C57BL/6 (Hopkins et al., 2019) and C3H/HeN mice, where LXR/RXR appears to be activated, suggesting differences in gene regulation between macrophages and epithelial cells. We postulate that *C. rodentium*‐induced colitis leads to establishment of a novel balance between cholesterol biogenesis and efflux that could impact on immune responses and the microbiome and thereby influence disease progression irrespective of the host genetic background.

We observed increased abundance of the pattern recognition receptors NLRP3 and ALPK1 specifically in IECs of C3H/HeN mice from 6 DPI, both at the transcriptional and translational levels. Activation of SREBP2 triggers expression and activation of NLRP3 via TIFA, whereas a SCAP‐SREBP2 complex in the ER has recently been shown to directly activate the NLRP3 inflammasome (Guo et al., 2018; Lin et al., 2016; Xiao et al., 2013). Whereas ALPK1 was induced specifically in C3H/HeN mice, TIFA was found in higher abundance in both C3H/HeN and C57BL/6 mice. This was expected given the reported TLR4/MYD88‐dependent TIFA upregulation in response to hypoxia‐reoxygenation of the liver; a phenomenon also undergone in the colon during *C. rodentium* infection regardless of host genetics (Ding et al., 2013).

Recent studies revealed that the *Hiccs*/*Cdcs1* locus‐encoded *Alpk1* plays a key role in regulating intestinal inflammation. A congenic 129SvEv strain carrying the B6 allele of the *Hiccs*/*Cdcs1* locus phenocopies the colitis resistance seen in C57BL/6 mice. Transcriptomic analysis of colon tissue from 129.*Rag2* ^*−/−*^ and 129.*Hiccs* ^*B6*^.*Rag2* ^*−/−*^ mice during the early phase of infection with the pathobiont *Helicobacter hepaticus* (*Hh*) revealed robust induction of inflammatory genes as early as 2 DPI in the former, which resembles the rapid response we observed following infection of C3H/HeN mice with *C. rodentium*. However, although ALPK1 regulates colitis via the haematopoietic compartment during *Hh* infection, we have shown here that upon *C. rodentium* infection of C3H/HeN mice, expression of ALPK1 is upregulated in IECs. At present, it is not known if *C. rodentium* infection triggers expression of ALPK1 in other cell types or whether it plays an active role in pathogen–host interaction in C3H/HeN mice. The cues and regulatory processes controlling expression of ALPK1 in C3H/HeN mice are also unknown. Importantly, higher abundance of ALPK1 mRNA is found in the inflamed intestinal mucosa of patients with IBD relative to tissue from healthy controls (Ryzhakov et al., 2018), further supporting the utilisation of *C. rodentium* infection of C3H/HeN as an IBD model (Mullineaux‐Sanders et al., 2019).

Activation of ALPK1‐induced NF‐κB signalling by the PAMP ADP‐hep has recently been reported in the intracellular pathogen *Shigella flexneri* (Garcia‐Weber et al., 2018) and the extracellular pathogens *Yersinia pseudotuberculosis* (Zhou et al., 2018) and *Helicobacter pylori* (Pfannkuch et al., 2019). Indeed, using a reporter cell line, we show activation of the ALPK1 pathway by ADP‐hep during *C. rodentium* infection in vitro. This activation was independent of the T3SS. As mutations in Δ*hldE* and Δ*rfaC* attenuated bacterial growth, we were unable to test the outcome of Δ*hldE* and Δ*rfaC* infection in vivo.

Taken together, our data show that in contrast to C57BL/6 mice, the infection phases of *C. rodentium* in mice suffering from severe disease are divided into (1) the caecal establishment phase (1 DPI), (2) the colonic expansion phase (2–3 DPI), (3) the shedding steady‐state phase (colonisation above 5 × 10^8^ CFU g^−1^ stool; 4–8 DPI), and (4) the host decline Phase 4 (from around 8 DPI), when the health status of the host rapidly declines. We show that robust mucosal responses to *C. rodentium* infection are rapidly induced in C3H/HeN mice during Phase 2 and demonstrate for the first time *C. rodentium*‐induced ALPK1 upregulation in vivo and ALPK1 activation in vitro. We propose that failure to clear the pathogen and consequent mortality of C3H/HeN mice is attributable to early and exaggerated pathway activation leading to irrecoverable tissue damage.

## 4 EXPERIMENTAL PROCEDURES

### 4.1 Bacterial strains

Bacterial strains used in this study are listed in Table S1. Strains were cultured at 37°C in Lysogeny broth (LB), with 200 rpm shaking, or on LB solidified agar (Merck; 3.7%). If required, nalidixic acid was added at 50 μg ml^−1^. Growth curves were carried out on a FLUOstar Omega plate reader. Bacteria grown to stationary phase were normalised to an OD_600_ of 0.1 in LB or minimal (M9) media supplemented with glucose (0.4%) or L‐lactate (0.4 or 0.8% w/v).

### 4.2 Generation of *C. rodentium* mutants

All plasmids and primers used are listed in Tables S2 and S3. pSEVA612s donor plasmid was generated via initial PCR amplification of gene of interest and flanking regions (using primers DC235 and DC236 for *hldE* and DC238 and DC239 for *rfaC*). Purified PCR amplicon (Qiagen) was digested in CutSmart buffer with High Fidelity enzymes (New England Biolabs [NEB]) and ligated into the pSEVA612s vector using T4 Ligase (NEB) as per manufacturer's recommendations. pSEVA612s derivatives were then chemically transformed into CC118λpir *E. coli* (Herrero, de Lorenzo, & Timmis, 1990). Inverse PCR was subsequently carried out on constructed plasmids (purified using Monarch® Plasmid Miniprep kit [NEB] as per manufacturer instructions), to obtain linearised pSEVA612s containing GOI flanking regions only (primers DC240 and DC241 for *hldE* and DC242 and DC243 for *rfaC* were used). Linearised DNA was dpnI (NEB) digested for 1 hr at 37°C and PCR purified. Purified amplicon was phosphorylated (Calf intestinal phosphatase, NEB) and ligated (T4 ligase, NEB) as per manufacturer recommendations and chemically transformed CC118λpir *E. coli* (Herrero et al., 1990). *hldE* and *rfaC* were individually deleted from the *C. rodentium* ICC169 (receiver) strain via tri‐parental conjugation using donor plasmid, pSEVA612s and helper plasmid, pRK2013, from *E. coli* strains CC118λpir and CC1047, respectively. Tri‐parental conjugation was carried out as previously described (Berger et al., 2017). All plasmids and deletion mutants were confirmed by sequencing (Eurofins). Deletion mutants were tested for growth attenuation by measuring OD600 during growth in LB at 37°C with 200 rpm shaking.

### 4.3 TIFA reporter cell line

HeLa cells (ATCC) were cultured in Dulbecco's modified Eagle's medium (DMEM) supplemented with 10% FCS and 2‐mM GlutaMAX‐1 (complete growth medium) at 37°C under 5% CO_2_. HeLa cells stably expressing TIFA‐GFP were obtained after transfection of pEGFP‐C1 plasmid encoding TIFA‐GFP and geneticin selection at 1 mg ml^−1^ (Garcia‐Weber et al., 2018).

### 4.4 Infection of HeLa cells

HeLa cells were seeded in 96‐well plates at 1.2 × 10^4^ cells/well and grown for 48 hr. Prior to infection, they were incubated with 100 μl of DMEM. For priming of *C. rodentium*, a saturated LB culture was diluted 1/500 in DMEM containing 1 g L^−1^ glucose and grown in a humidified incubator at 37°C, 5% CO_2_ without agitation overnight. Bacteria were washed and added to cells at indicated MOIs. In control conditions, cells were treated or not with 10^−7^ M ADP‐hep (J&K Scientific). Plates were then centrifuged at 500 *× g* for 5 min to synchronise infection and incubated at 37°C, 5% CO_2_. After 3 hr, cells were fixed for 15 min with 4% phosphate buffered saline (PBS)‐paraformaldehyde, washed in PBS (*×*3), and then processed for immunostaining.

### 4.5 Immunostaining

Cells were permeabilised with 0.1% Triton×100 (Sigma) for 5 min at room temperature and incubated with a primary rabbit polyclonal anti‐*C. rodentium* antibody (1/200) for 1 hr. They were then washed in PBS and incubated with a donkey anti‐rabbit Alexa Fluor 467 antibody (1/500, Invitrogen) for *C. rodentium* staining. DNA was stained with Hoescht 33342. Images were acquired with an ImageXpress Micro (Molecular Devices, Sunnyvale, USA). Each data point corresponds to duplicate wells; nine images were taken per well. Image analysis was performed using the custom module editor of MetaXpress as previously described (Garcia‐Weber et al., 2018). For quantification of TIFA‐GFP oligomerisation, TIFAsomes were detected by applying the “Find blobs” function based on size and intensity above background parameters. Cell nuclei were identified with the “Find round objects” module. Cells were defined by extending each nucleus by 10 pixels with the “Grow objects without touching” function, and TIFA punctates were quantified within this mask.

### 4.6 Ethics statement

All animal experiments complied with the Animals Scientific Procedures Act 1986 and U.K. Home Office guidelines and were approved by the Animal Welfare and Ethical Review Body at Imperial College London. Experiments were designed in agreement with the ARRIVE guidelines for the reporting and execution of animal experiments, including sample randomisation and blinding (Kilkenny et al., 2010).

### 4.7 Infection of mice

Pathogen‐free female 8‐ to 10‐week‐old C3H/HeNCrl mice (Charles River Laboratories) were housed in high‐efficiency particulate air‐filtered cages, with food and water given ad libitum. Mouse experiments were performed with a minimum of four mice per group. Mice were orally gavaged as described (Crepin, Collins, Habibzay, & Frankel, 2016). Uninfected mice were given PBS (200 μl). Enumeration of viable bacteria (CFU) per gram of stool sample was determined by plating onto selective agar as previously described (Crepin et al., 2016). Faecal water content was determined by weighing stools pre and post vacuum drying.

### 4.8 Bioluminescent imaging

Depilated mice were imaged using the IVIS® Spectrum CT (Perkin Elmer) system under gaseous anaesthesia with isofluorane (Zoetis). Images were processed using Living Image software (Perkin Elmer). For BLI of tissue at necropsy, the colon and ceacum were removed and cut longitudinally, and the ceacal contents and stools were removed. The colonic and ceacal tissue were washed briefly in PBS and the tissues imaged individually, with the mucosa exteriorised in an IVIS Spectrum CT.

### 4.9 Tissue staining

The terminal 0.5‐cm distal colon was fixed in 4% paraformaldehyde solution (diluted in PBS) for 2 hr and paraffin embedded. Paraffin embedded sections were treated for heamotoxylin and eosin (H&E; Crepin et al., 2016). AB/PAS staining was carried out according to standard techniques (Hopkins et al., 2019). For immunohistochemistry, samples were treated with primary antibodies, mouse PCNA (1:500, Abcam), chicken anti‐intimin (1:50, a gift from Professor Fairbrother, Montreal University), and secondary antibodies from Jackson ImmunoResearch (all 1:200). DNA was counter‐stained with Hoechst 33342. Images were acquired using a Zeiss AxioVision Z3 microscope, using an AxioCam MRm camera and processed using Zen 2.3 (Blue Version; Carl Zeiss MicroImaging GmbH, Germany).

### 4.10 Extraction of IECs

IECs were extracted from 4‐cm distal colon as previously described (Berger et al., 2017) and frozen at −80°C until processing for proteomic analysis or RNA extraction.

### 4.11 Mass spectrometry

IEC samples were processed alongside a CMT‐93 cell line pellet as an internal control. All samples were dissolved in 100 μl of 0.1 M triethylammonium bicarbonate, 0.1% SDS, 10% isopropanol on ice, assisted with pulsed probe sonication. Samples were then boiled at 90°C for 5 min on a shaking device at 300 rpm. Protein concentration was measured with Quick Start Bradford protein assay (Bio‐Rad) according to manufacturer's instructions. Aliquots containing 100 μg of total protein were prepared for trypsin digestion. Samples were reduced with 5 mM of tris‐2‐carboxyethyl phosphine and alkylated with iodoacetamide, followed by adding 75 ng μl^−1^ trypsin to each sample and leaving at RT to incubate overnight. The resulting peptides were diluted up to 100 μl with 0.1 M of triethylammonium bicarbonate buffer and labelled with TMT 11‐plex reagent vial (ThermoFisher Scientific) according to manufacturer's instructions. Hydroxylamine was used to quench the reaction, and then all 11 samples were combined in equal amounts to a single tube. The sample was then dried with a centrifugal vacuum concentrator. Basic reverse‐phase peptide fractionation, LC‐ESI‐MS/MS analysis, and database search were carried out as previously described on an Orbitrap Fusion (Thermo) using the Synchronous Precursor Selection method (Hopkins et al., 2019). For protein quantification, only unique peptides with an average reported signal‐to‐noise >3 were used. The values obtained from uninfected mice were used as baseline controls for Log2 ratio calculations for WT infected mouse samples carried out in duplicate. The mass spectrometry proteomics data have been deposited to the ProteomeXchange Consortium via the PRIDE partner repository with the dataset identifier PXD012846.

### 4.12 Data analysis

KEGG and GOBP slim pathway enrichment analysis was performed with the 1D‐enrichment method (Cox & Mann, 2012). The terms were subsequently filtered for Benjamini–Hochberg FDR <0.05. Ingenuity pathway analysis (Qiagen) platform was used for construction and visualisation of cellular pathways.

### 4.13 RNA extraction and quantitative real‐time RT‐PCR

RNA was extracted from IEC pellets using the RNeasy® Mini kit (Qiagen) according to manufacturer's instructions. RNA samples were RQ1 DNase (Promega) treated. Reverse transcription to cDNA was carried out using Moloney murine leukaemia virus reverse transcriptase (Promega) according to the manufacturer's recommendations. Amplification from cDNA was performed using Power SYBR® Green PCR Master Mix (ThermoFisher Scientific). Assay reactions were performed in a 20‐μl volume with 2× Power SYBR® Green PCR Master Mix, efficiency optimised primers to a final concentration of 0.05 μM each and cDNA. All experiments included nontemplate controls and were carried out in technical replicate with a minimum of four biological repeats. The ΔΔC_t_ method of quantification was performed to give Log2 FC relative to the expression of averaged baseline measurement (Livak & Schmittgen, 2001). Expression was normalised to the housekeeping gene *Gapdh*. Primer pairs are as previously reported (Hopkins et al., 2019). Additional primer pairs are listed in Table S4.

### 4.14 Colorimetric cholesterol assay

Stools were collected from mice, vacuum dried, and weighed before being crunched to powder and lipids extracted in an 800‐μl mixture of chloroform : isopropanol : NP‐40 (7:11:0.1). Faecal cholesterol was quantified using a colorimetric reaction, as per the manufacturer's recommendations (Cell Biolabs, STA‐384). Colorimetric signal was analysed using the FLUOstar Omega spectrophotometric microplate reader (BMG Labtech) in the 540‐ to 570‐nm range.

### 4.15 16S rRNA gene sequencing

Upon necroscopy, 1‐cm distal colon was immediately frozen and stored at −80°C. DNA was extracted using a Power Soil kit (MoBio). The 16 s V4 region was amplified using 515F/806R primers and sequenced using 2 × 250 bp paired‐end sequencing (Illumina MiSeq). Sequences were analysed using the Qiime (Quantitative Insights into Microbial Ecology, http://www.qiime.org) analysis pipeline (Caporaso et al., 2010).

### 4.16 Statistical analysis

Graphpad Prism 7 was used for all statistical calculations unless otherwise specified. FC values were log transformed prior to analysis of variance being carried out. *P* values <.05 were considered significant (^*^ *P* ≤ .05, ^**^ *P* ≤ .01, ^***^ *P* ≤ .001, and ^****^ *P* ≤ .0001). Statistical analysis of the microbiome was performed at individual taxon levels. For each taxon, the Mann–Whitney *U* statistical test was carried out between uninfected and infected groups, correcting *P* values for FDR using the Benjamini and Hochberg method. Unpaired *t* test was used for statistical analysis of faecal water content and quantification of total bioluminescent flux from ex vivo tissues. Pathway analysis was performed with the 1D enrichment method in the Perseus software (Cox & Mann, 2012; Tyanova et al., 2016). The enrichment score shows whether the proteins of a pathway tend to be upregulated or downregulated based on Wilcoxon–Mann–Whitney test and is normalised within min = −1 and max = 1. Comparison to C57BL/6 proteins at 8 DPI was carried out using data deposited in PRIDE with identifier PXD005004 (Berger et al., 2017). Proteins from enriched pathways of interest (Benjamini–Hochberg FDR < 0.05) were selected based on previous literature on C57BL/6 mice. Proteins were considered significant if abundance changed 1.5‐fold or higher upon infection.

## AUTHOR CONTRIBUTIONS

DC performed the experiments, analysed the data, and wrote the manuscript. EGDH performed the proteomics aspects, analysed the data, and edited the manuscript. TIR was involved in preparing and running the mass spectrometry samples, data analysis, and editing the manuscript. CMS performed the microbiome analysis, with the help of EE, and edited the manuscript. RB supervised the project, analysed the data, and edited the manuscript. DGW and CA designed and performed the in vitro cell infection assays and edited the manuscript. GF and JSC were responsible for overseeing the overall running of the project, data analysis, and writing the paper.

## CONFLICT OF INTERESTS

The authors declare no conflict of interest.

## Supporting information

Table S1. Strains used in this study

Figure S1. BLI of *C. rodentium* infection in C57BL/6 mice
**A.** *In vivo* images of C57BL/6 mice infected with *C. rodentium* ICC180 at 4‐8 DPI (n=5), showing mainly caecal colonisation at 4 DPI and colonic colonisation from 5 DPI. The scale bar indicates signal intensity (photons s−1 cm−2 sr−1). Total flux as measured *ex vivo* from the caecum (**B**) and colon (**C**) at 8 DPI. **=*p*<0.01, ****=< *p*<0.0001 (Student's *t*‐test).

Figure S2. Quantification of *C. rodentium* shedding
CFU/g stool measurements taken from C3H/HeN (**A**) and C57BL/6 (**B**) mice prior to IEC extraction for proteomics analysis. In order to reduce heterogeneity within conditions, the mouse that did not reach the 1 x 10^8^ CFU/g stool at 6 DPI cut‐off was excluded from the experiment (as indicated by dotted line).

Figure S3. Comparison of protein enrichment following *C. rodentium* infection of C3H/HeN and C57BL/6
**A**. A comparison of enriched KEGG pathways following *C. rodentium* infection in C3H/HeN and C57BL/6 mice as determined by 1D enrichment analysis revealed largely conserved changes to processes between hosts.

Figure S4. Genus level analysis of Phlya significantly changed upon *C. rodentium* infection of susceptible mice.
**A.** Weighted and unweighted principle coordinates analysis (PCoA) of uninfected (n=4) and ICC169 infected tissue (n=5). Graphs indicating Gammaproteobacteria (**B**), Firmicutes (**C**), Actinobacteria (**D**), Verrucomicrobia (**E**), Defferibacteres (**F**) and Bacteroidetes (**G**) significantly changed upon *C. rodentium* infection at genus level (unless otherwise stated). For uninfected samples, n=4 and for infected samples, n=5, *p*< 0.05 (Mann‐Whitney with FDR correction).

Figure S5. Attenuation of Δ*hldE* and Δ*rfaC* mutants
**A.** Schematic of LPS inner core biosynthesis pathway, genes targeted for mutation are indicated by red box. Growth kinetics of WT *C. rodentium*, Δ*hldE* and Δ*rfaC* mutants in LB (**B**) and M9 minimal (**C**) media revealing significant growth attenuation of the mutant. ****=< *p*<0.0001 (one‐way ANOVA).

Figure S6. Quantification of TIFAsomes
**A.** Quantification of TIFAsomes following 1,2 or 3 h infection at MOI 100 with WT, Δ*escN,* Δ*hldE,* Δ*rfaC C. rodentium* or treatment with ADP‐hep control. Data correspond to the mean + SD of three experiments, *=*p*≤0.05, , ****=*p*≤0.0001 (two‐way ANOVA).
