# Supplementary material for: Citrobacter rodentium induces rapid and unique metabolic and inflammatory responses in mice suffering from severe disease
Source: Cell Microbiol. 2019 Oct 30;22(1):e13126. doi: 10.1111/cmi.13126 (PMC7003488; doi:10.1111/cmi.13126)
Supplement: Supplementary file 1 — Table S1. Strains used in this study [file CMI-22-e13126-s001.docx]

**Table S1. Strains used in this study**

| **Strain** | **Source** | **Identifier** |
| --- | --- | --- |
| Wild type Nal^R^ derivative of *C. rodentium* ICC168 | [65] | ICC169 |
| *Salmonella* serovar Typhimurium | [66] | ICC797 |
| ICC169 Δ*hldE* | This study | ICC2032 |
| ICC169 Δ*rfaC* | This study | ICC2033 |

**Table S2. Plasmids used in this study**

| **Plasmid** | **Identifier** | **Resistance** | **Source** |
| --- | --- | --- | --- |
| pSEVA612s *hldE flanking regions* | pICC2765 | Gm | This study |
| pSEVA612s *rfaC flanking regions* | pICC2766 | Gm | This study |
| pACBSR | N/A | Sm | [67] |
| pRK2013 | N/A | Kn | [68] |

**Table S3. Cloning primers used in this study**

| **Primer name** | **GOI** | **Restriction enzyme** | **Direction** | **Sequence** |
| --- | --- | --- | --- | --- |
| DC235 | *hldE* | SphI | Fwd | TACTGCATGCGGCGGCTGCCATTGTGGAAC |
| DC236 | *hldE* | SacI | Rev | TACTGAGCTCCCGCAGTTAACCGCGCAGTTC |
| DC238 | *rfaC* | SacI | Rev | TACTGCATGCCCTTTATTATAATCTTGCGCGTACAGAAATAGTTCGAC |
| DC239 | *rfaC* | SalI | Fwd | TACTGTCGACCCTTTATTATAATCTTGCGCGTACAGAAATAGTTCGAC |
| DC240 | *hldE* | KpnI | Fwd | ACAAGGTACCACGTCCTGTAAAGTGCCGGATGGCGCTGCG |
| DC241 | *hldE* | BamHI | Rev | CAAGGGATCCTCCTGTCTCCTGAGAGATTCAAAATTTGCG |
| DC242 | *rfaC* | KpnI | Fwd | ACAAGGTACCAAACCCATTTCGCTTCGCCATATTGTGGAG |
| DC243 | *rfaC* | BamHI | Rev | CAAGGGATCCTCGTCAGGCTTCCTCTTGTAATAACAGGC |

**Table S4. qRT-PCR primers used in this study**

| **Primer name** | **GOI** | **Direction** | **Sequence** |
| --- | --- | --- | --- |
| DC174 | *Nlrp3* | Fwd | GTTTTCCCAGACACTCATGTTG |
| DC175 | *Nlrp3* | Rev | GTCTCCCAGAGTATTGTCACTG |
| DC256 | *Slc13a3* | Rev | GGCACTCAGAAGTTCTCCAG |
| DC257 | *Slc13a3* | Fwd | CCGCCAGGATGAACACATAC |
| DC258 | *Ldha* | Fwd | GCTCCCCAGAACAAGATTACAG |
| DC259 | *Ldha* | Rev | TCGCCCTTGAGTTTGTCTTC |
