# Supplementary figures and images for: Citrobacter rodentium induces rapid and unique metabolic and inflammatory responses in mice suffering from severe disease
Source: Cell Microbiol. 2019 Oct 30;22(1):e13126. doi: 10.1111/cmi.13126 (PMC7003488; doi:10.1111/cmi.13126)

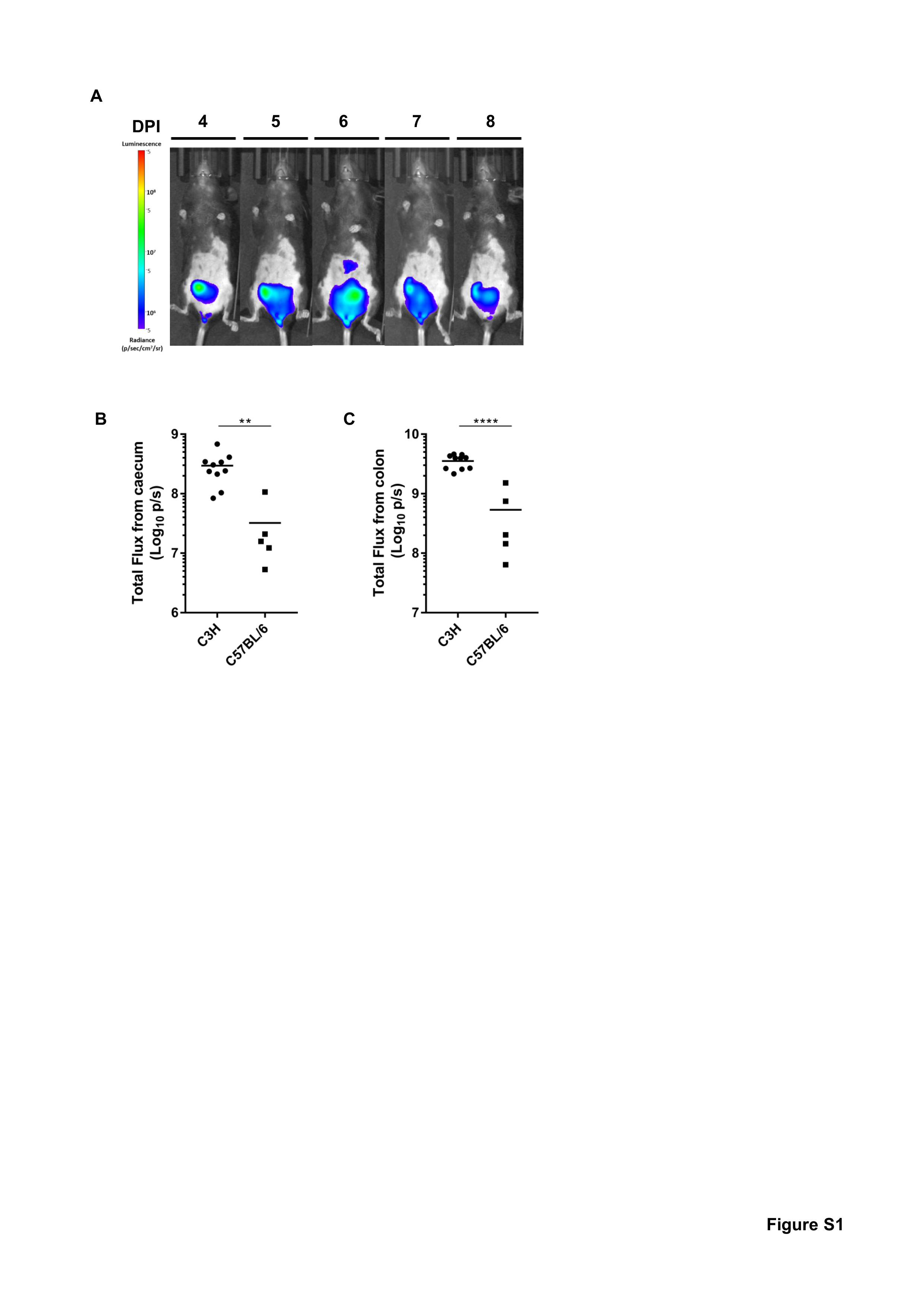

Supplement: Supplementary file 2 — Figure S1. BLI of C. rodentium infection in C57BL/6 mice A. In vivo images of C57BL/6 mice infected with C. rodentium ICC180 at 4‐8 DPI (n=5), showing mainly caecal colonisation at 4 DPI and colonic colonisation from 5 DPI. The scale bar indicates signal intensity (photons s−1 cm−2 sr−1). Total flux as measured ex vivo from the caecum (B) and colon (C) at 8 DPI. **=p<0.01, ****=< p<0.0001 (Student's t‐test). [file CMI-22-e13126-s002.tif]

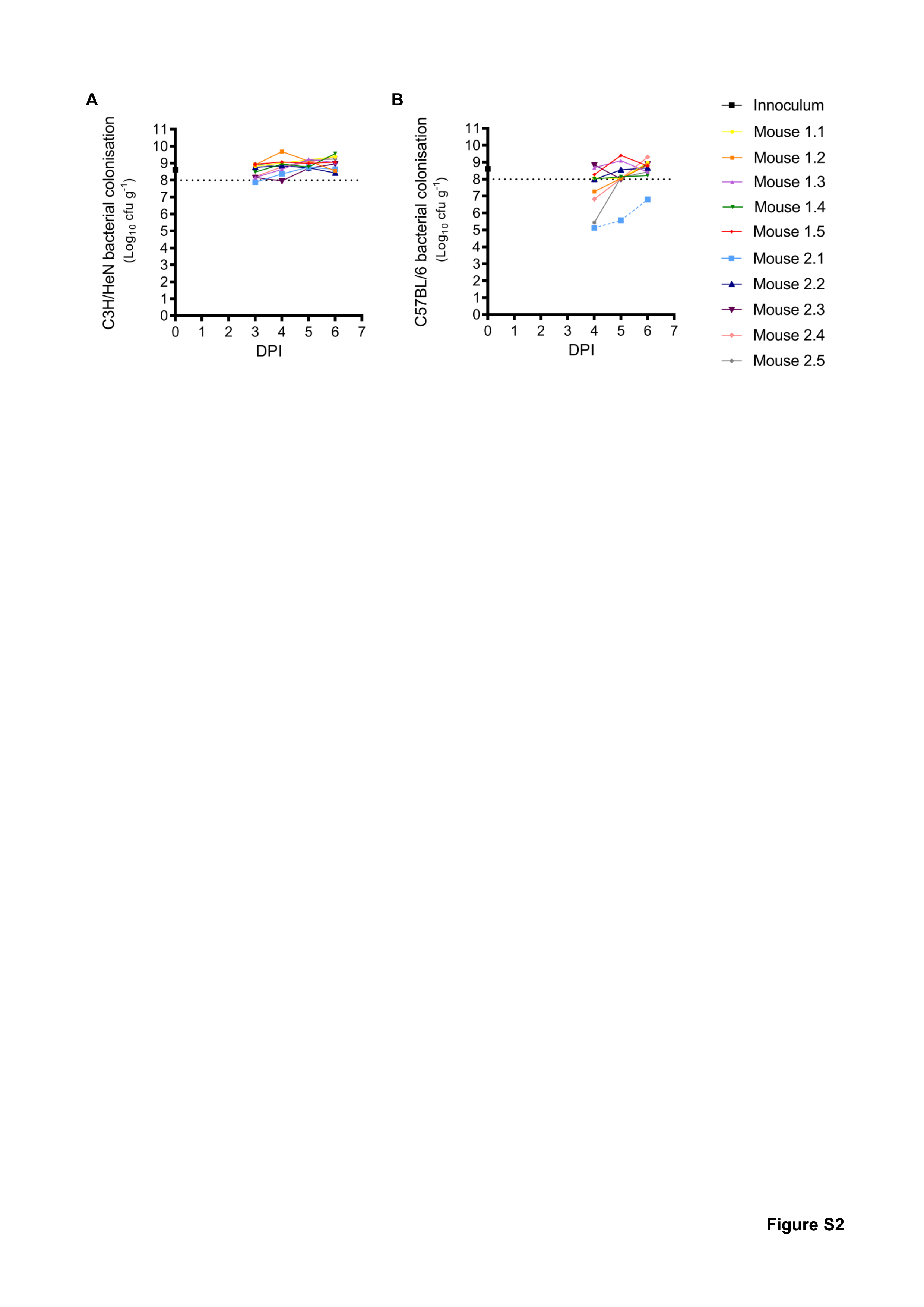

Supplement: Supplementary file 3 — Figure S2. Quantification of C. rodentium shedding CFU/g stool measurements taken from C3H/HeN (A) and C57BL/6 (B) mice prior to IEC extraction for proteomics analysis. In order to reduce heterogeneity within conditions, the mouse that did not reach the 1 x 10^8^ CFU/g stool at 6 DPI cut‐off was excluded from the experiment (as indicated by dotted line). [file CMI-22-e13126-s003.tif]

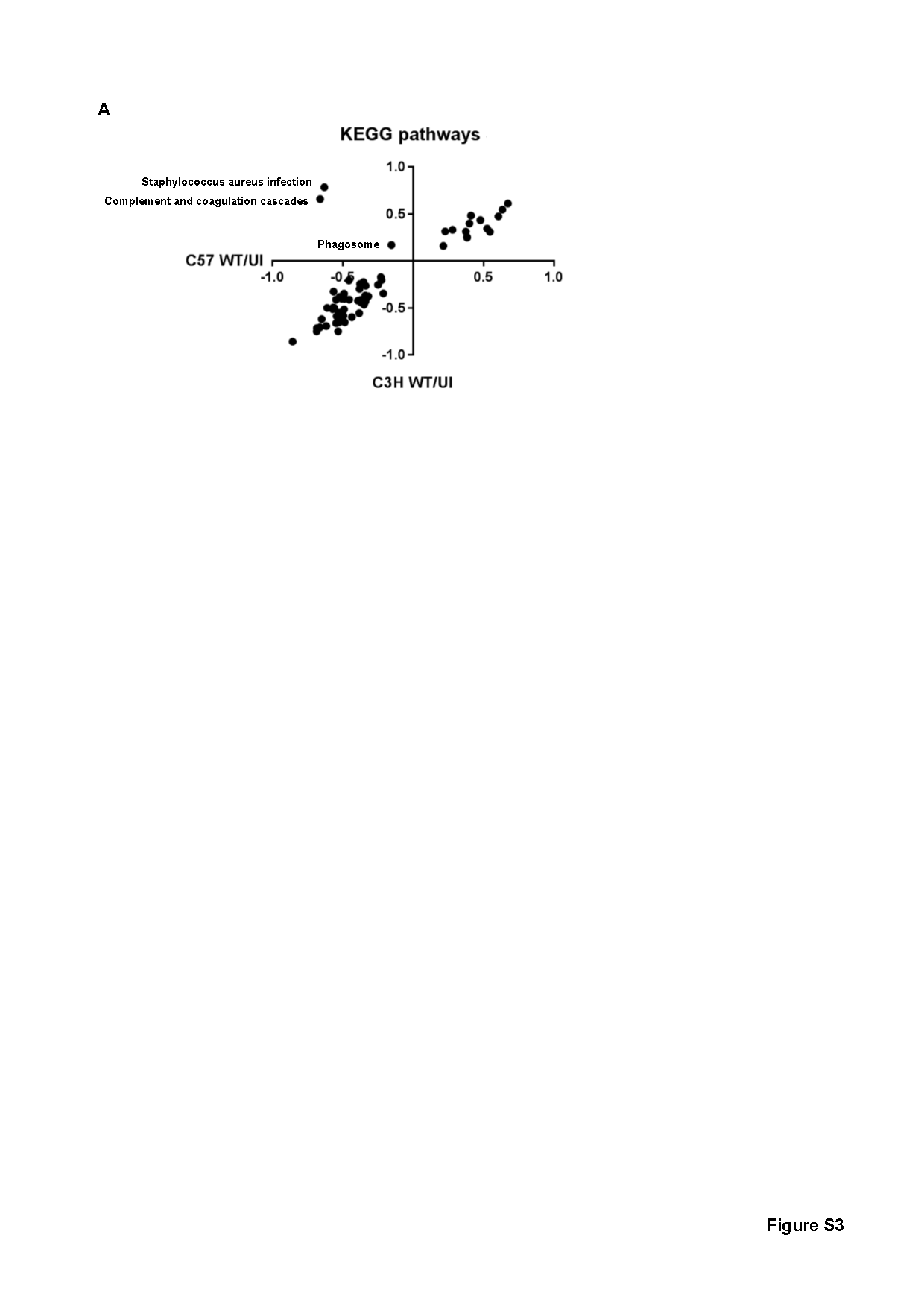

Supplement: Supplementary file 4 — Figure S3. Comparison of protein enrichment following C. rodentium infection of C3H/HeN and C57BL/6 A. A comparison of enriched KEGG pathways following C. rodentium infection in C3H/HeN and C57BL/6 mice as determined by 1D enrichment analysis revealed largely conserved changes to processes between hosts. [file CMI-22-e13126-s004.tif]

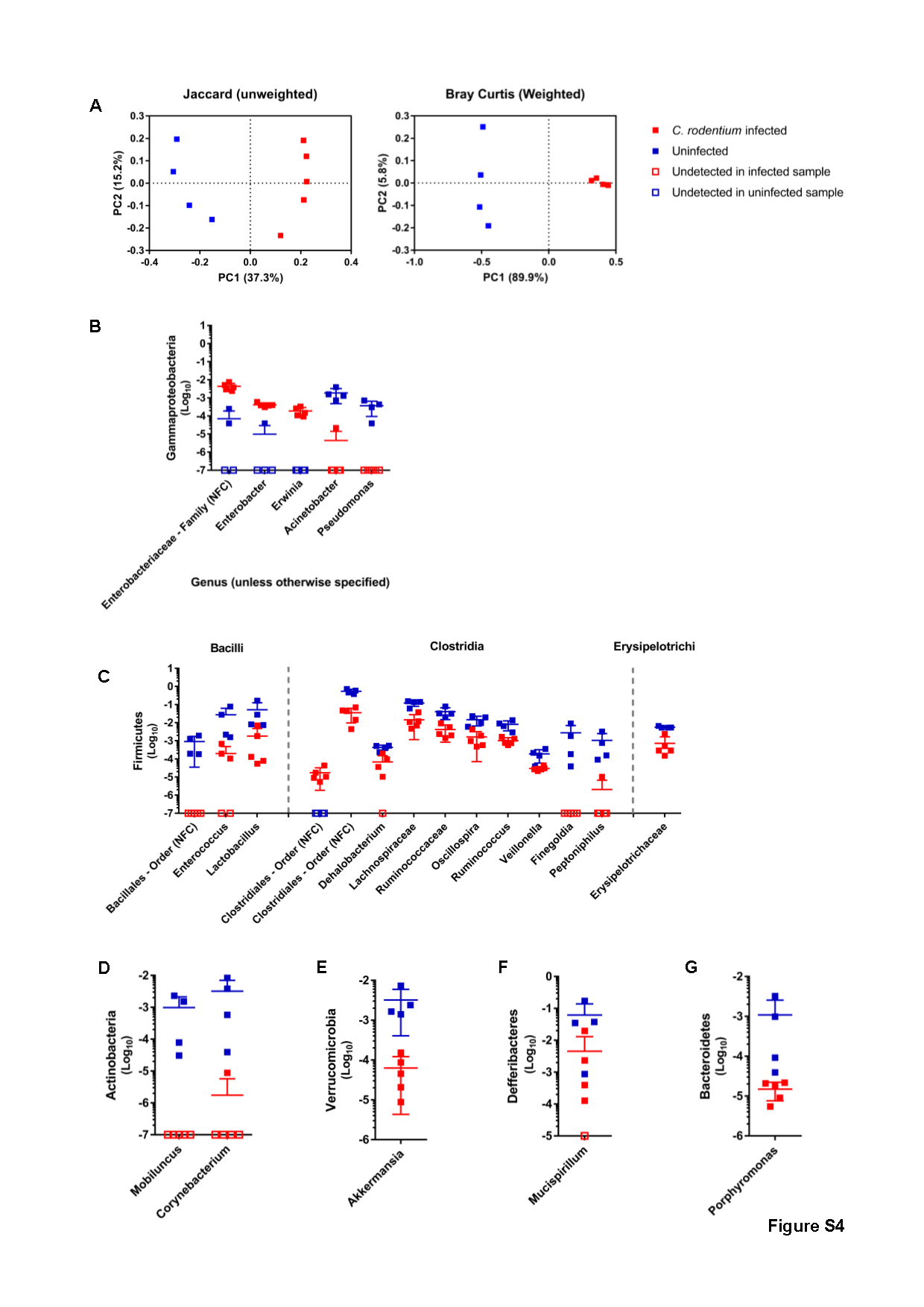

Supplement: Supplementary file 5 — Figure S4. Genus level analysis of Phlya significantly changed upon C. rodentium infection of susceptible mice. A. Weighted and unweighted principle coordinates analysis (PCoA) of uninfected (n=4) and ICC169 infected tissue (n=5). Graphs indicating Gammaproteobacteria (B), Firmicutes (C), Actinobacteria (D), Verrucomicrobia (E), Defferibacteres (F) and Bacteroidetes (G) significantly changed upon C. rodentium infection at genus level (unless otherwise stated). For uninfected samples, n=4 and for infected samples, n=5, p< 0.05 (Mann‐Whitney with FDR correction). [file CMI-22-e13126-s005.tif]

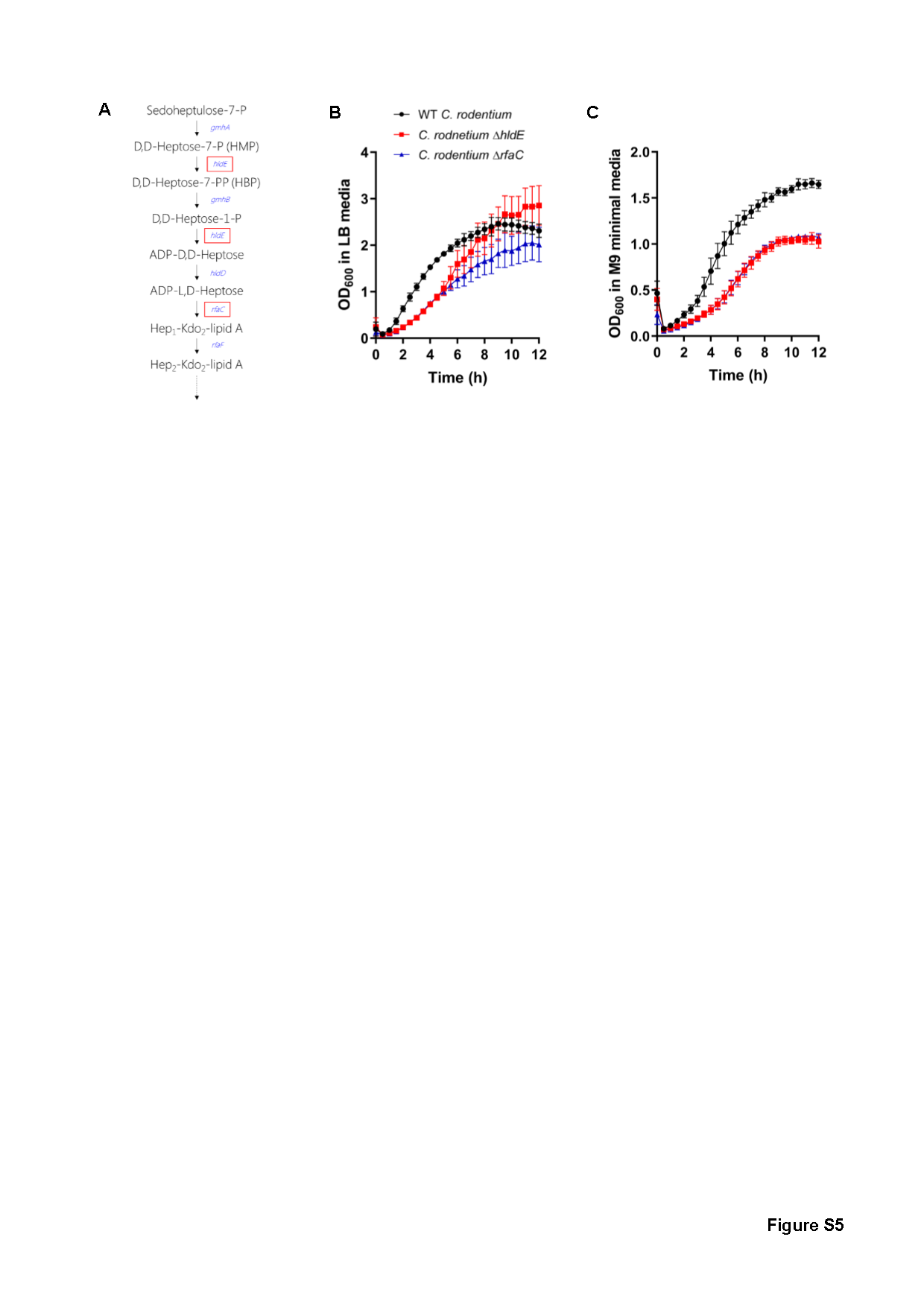

Supplement: Supplementary file 6 — Figure S5. Attenuation of ΔhldE and ΔrfaC mutants A. Schematic of LPS inner core biosynthesis pathway, genes targeted for mutation are indicated by red box. Growth kinetics of WT C. rodentium, ΔhldE and ΔrfaC mutants in LB (B) and M9 minimal (C) media revealing significant growth attenuation of the mutant. ****=< p<0.0001 (one‐way ANOVA). [file CMI-22-e13126-s006.tif]

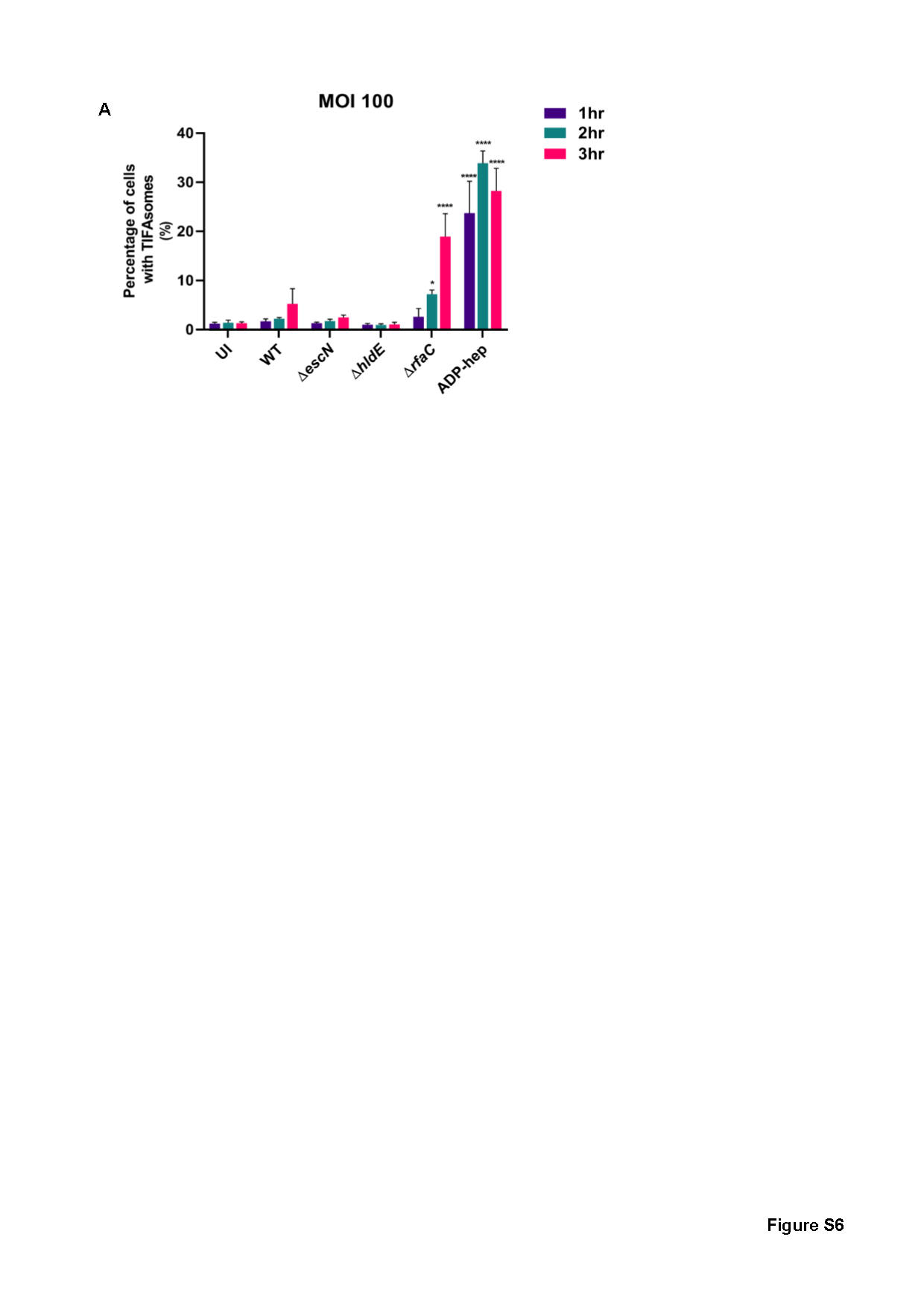

Supplement: Supplementary file 7 — Figure S6. Quantification of TIFAsomes A. Quantification of TIFAsomes following 1,2 or 3 h infection at MOI 100 with WT, ΔescN, ΔhldE, ΔrfaC C. rodentium or treatment with ADP‐hep control. Data correspond to the mean + SD of three experiments, *=p≤0.05, , ****=p≤0.0001 (two‐way ANOVA). [file CMI-22-e13126-s007.tif]
